# Factors influencing clinical pharmacists’ integration into the clinical multidisciplinary care team

**DOI:** 10.3389/fphar.2023.1202433

**Published:** 2023-06-12

**Authors:** Chenyu Wang, Maozhen Li, Yuankai Huang, Xiaoyu Xi

**Affiliations:** National Medical Products Administration Key Laboratory for Drug Regulatory Innovation and Evaluation, China Pharmaceutical University, Nanjing, China

**Keywords:** clinical pharmacist, multidisciplinary care team, interprofessional collaboration, influencing factors, China

## Abstract

**Objectives:** To investigate the factors influencing clinical pharmacists’ integration into the clinical multidisciplinary care team, using interprofessional collaboration between clinical pharmacists and physicians as the focus.

**Methods:** Through stratified random sampling, a cross-sectional questionnaire survey was conducted among clinical pharmacists and physicians in secondary and tertiary hospitals in China from July to August 2022. The questionnaire, comprising the Physician–Pharmacist Collaborative Index (PPCI) scale to reflect the collaboration level and a combined scale to measure influencing factors, was made available in two versions for clinical pharmacists and physicians. Multiple linear regression was adopted to analyze the association between the collaboration level and influencing factors, as well as the heterogeneity of the significant factors in hospitals of different grades.

**Results:** Valid self-reported data from 474 clinical pharmacists and 496 paired physicians were included, who were serving in 281 hospitals from 31 provinces. In terms of participant-related factors, standardized training and academic degree, respectively, exerted significant positive effects on the perceived collaboration level by clinical pharmacists and physicians. In terms of context characteristics, manager support and system construction were the main factors for improving collaboration. In terms of exchange characteristics, clinical pharmacists having good communication skills, physicians trusting others’ professional competence and values, and both parties having consistent expectations had significant positive effects on collaboration.

**Conclusion:** The study provides a baseline data set on the current level and associated factors of clinical pharmacists’ collaboration with other professionals in China and other countries with a related health system, providing references for individuals, universities, hospitals, and national policymakers to facilitate the development of clinical pharmacy and multidisciplinary models and further improve the patient-centered integrated disease treatment system.

## 1 Introduction

Clinical pharmacotherapy requires a patient-centered approach to minimize medication risks while improving patient health outcomes (Hepler and Strand, 1990). However, due to the increasing disease complexity and drug diversification, avoiding clinical medication errors has become a global challenge. Currently, establishing multidisciplinary or interprofessional teams is widely respected to integrate the resources and strengths of various medical personnel to improve the quality of treatment and care, and pharmacists are also encouraged to participate in these teams and activities (Billstein-Leber et al., 2018). Studies have shown that on the strength of their knowledge structure and professional sensitivity, clinical pharmacists pay more attention to the prevention of adverse drug reactions and the improvement of rationality, safety, and economy of drug use, especially in difficult and complex cases, by performing drug reconstitution, pharmaceutical care, and medication consultation and guidance and participating in treatment decision-making and scientific research (Hughes et al., 2017; Mcconnell et al., 2019).

Thus, the significance and value of integrating clinical pharmacists into multidisciplinary teams is becoming increasingly clear, and among them, the most critical is how to establish and facilitate the interprofessional collaboration between clinical pharmacists and physicians. In the United States, qualified pharmacists can enter into collaborative practice agreements (CPAs) with physicians to specify responsibilities and be granted limited prescription authority for collaborative drug therapy management (CDTM). But it is not an easy task under the current healthcare system in China. First, the legal and regulatory systems related to pharmacists in China are not mature enough to form long-term perfect management mechanisms of collaboration and coordinate the competence and authority of various professionals, resulting in a vague role for clinical pharmacists. Although clinical pharmacists are gradually shifting their priority of work from basic drug dispensing to clinical services, decisions on patients’ drug treatment are absolutely dominated by physicians. Second, the cultivation of clinical pharmacy talents starts late in China, and clinical pharmacists are insufficient to meet the requirements of high professional skills and comprehensive quality to provide guidance and services to doctors, nurses, and patients, as well as to gain their trust. Moreover, the depth and breadth of collaboration always varies by the region (Mishra and Thomas, 2017).

To improve the collaboration between clinical pharmacists and physicians, a number of international studies have been conducted to investigate the facilitators or barriers, but the consistency and systematicity of the findings are limited due to differences in methods and scope of the studies (Van et al., 2011; Bechet et al., 2016; Alhossan and Alazba, 2019). Also, the applicability of these findings have to be further verified in China and other countries or regions where the collaboration model is still at the stage of theory-guided exploration, standard operating procedures for interprofessional collaboration in clinical multidisciplinary teams have not been promulgated, collaboration level varies from hospital to hospital, and few theoretical research and empirical evidence have been related to the influencing factors of collaboration (Zhu et al., 2010).

Therefore, this study intends to systematically sort out the influencing factors of clinical pharmacist–physician collaboration involved in existing studies and verify their actual effects on the collaboration under the current healthcare system in China through empirical research, so as to provide references and suggestions for policymakers to improve collaboration, promote better integration of the clinical pharmacist into the clinical team, and optimize the patient-centered integrated disease treatment system.

## 2 Materials and methods

### 2.1 Study design

A nation-wide cross-sectional questionnaire survey was conducted from 1 July to 31 August 2022. Stratified random sampling was used to take samples from clinical pharmacists and physicians from secondary and tertiary hospitals: a) Thirty-one provinces (autonomous regions and municipalities directly under the central government) in China were included, and all cities in each province were divided into three groups of high, medium, and low *per capita* disposable income in 2021, totaling 93 groups; b) The number of secondary and tertiary hospitals drawn from each group was determined by the average number of hospitals there were with reference to the annual health statistics yearbook (2021) released by Health Committees; c) According to the principle of convenient sampling, at least two clinical pharmacist questionnaires and two paired physician questionnaires were collected from each sample hospital.

### 2.2 Data collection

The study required that the clinical pharmacists and physicians participating in the survey should have experience in clinical pharmacotherapy collaboration, so the questionnaire collection method was such that after a clinical pharmacist filled in the questionnaire, the physician who had collaborative work experience with him/her was recommended to complete the questionnaire, to thereby ensure that the data would reflect the collaboration level in the hospitals comprehensively and truly from the perspectives of both the clinical pharmacists and physicians.

Based on the aforementioned requirements, this study recruited more than 400 undergraduate students from the school of pharmacy as investigators after training. With a mobile terminal in hand, the investigators conducted face-to-face research, explaining the nature, purpose, requirements, and precautions of the survey to the respondents after obtaining their consent. To ensure the authenticity and reliability of the research process and results, two data auditors were set up to initially audit the rationality of the research process and quality of filling the questionnaire in time.

### 2.3 Variables and measurement instructions

#### 2.3.1 Collaboration level

The Physician–Pharmacist Collaborative Index (PPCI) scale that was developed by Zillich et al., 2005; Zillich et al., 2006 for pharmacists and physicians, respectively, and has been validated and applied in several countries at different clinical pharmacy development levels (Sellappans et al., 2015; Al-Jumaili et al., 2017; Gemmechu and Eticha, 2020) was chosen as the tool to measure the collaboration level. It consists of 14 items divided into 3 dimensions: trustworthiness (6 items), role perception (5 items), and relationship initiation (3 items). Trustworthiness means the physician’s trust in the pharmacist’s professional competence and two-way commitment and communication; role perception focuses on mutual dependence and agreement on each other’s responsibilities; and relationship initiation refers to one party acting on the other’s needs to facilitate relationship progress. The sum of each item score, which is rated by using the 7-point Likert scale (1 = strongly disagree, 7 = strongly agree), reflects the physician’s and pharmacist’s perceptions of the current progress of the collaboration relationship.

In this study, two versions of the scale were translated and back-translated by two graduate students majoring in English with medical policy background to consider the actual situation in China and was further revised through expert consultation.

#### 2.3.2 Influencing factors

To comprehensively summarize the influencing factors of collaboration, this study refers to the collaborative working relationship (CWR) model that was first proposed by McDonough and Doucette (2000) and has been the main reference for subsequent collaborative theoretical models (Dey and de VMJWBosnic-Anticevich, 2011; Van et al., 2012; Van et al., 2013), classifying the influencing factors into three categories: participant characteristics, context characteristics, and exchange characteristics. The participant characteristics refer to the basic information of both pharmacists and physicians; context characteristics refer to the structures and conditions related to the collaboration; and exchange characteristics reflect the interaction tendencies between pharmacists and physicians. This classification framework has been widely used in studies related to factors influencing the pharmacist–physician collaboration and validated in countries with different levels of development (Van et al., 2011; Al-Jumaili et al., 2017; Nasir et al., 2020). Therefore, in this study, the three-characteristic classification framework was adopted to organize the possible influencing factors of collaboration.

**FIGURE 1:**
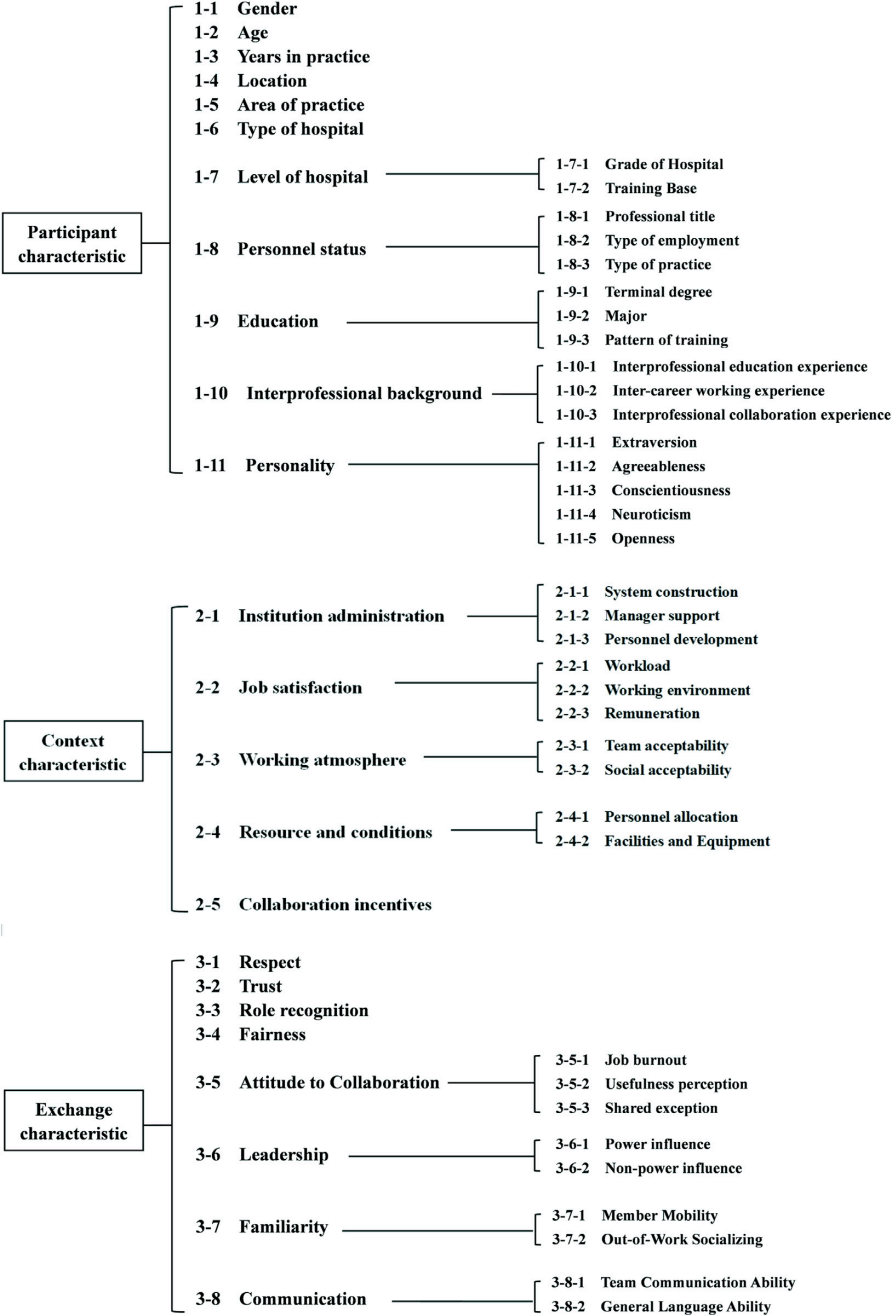
Framework of possible influencing factors.

Based on a systematic review and expert consultation, we summarized the influencing factors that may be relevant to collaboration and categorized them according to the aforementioned framework (Wang et al., 2022). The framework of possible influencing factors is shown in Figure 1 (the literature review process and specific connotations of each influencing factor are shown in Supplementary Material S1).

The measurement tool of influencing factors was a self-combined questionnaire. For factors that can be measured directly, objective questions were set after expert consultation; for factors that cannot be measured directly, the study referred to existing scales that met the following requirements: the measurement contents were consistent with the required connotation of the factors in this study, had been applied in the healthcare field in China or have universality in various fields of population; and have good reliability and validity. Considering the length and filling compliance of the questionnaire, the selected items were made as concise as possible, giving priority to the suitable scales with short form (see Table 1 in Supplementary Material S1 for the selection of scales for each factor). These scales without the Chinese version were also translated to preliminarily combine into the questionnaire on influencing factors of collaboration. The questionnaire also had two versions for clinical pharmacists and physicians, with few adjustments and deletions in the questions' formulation and option settings between them.

**TABLE 1:** Distribution of sample hospitals.

Category	N (%)
Grade of hospital	
Tertiary hospital	157 (55.87)
Secondary hospital	124 (44.13)
Location^1^	
Eastern region	122 (43.42)
Central region	72 (25.62)
Western region	87 (30.96)
Type of hospital	
General hospital	240 (85.41)
Specialized hospital	41 (14.59)

Note: ^1^The eastern region includes Beijing, Shanghai, Tianjin, Hebei, Jiangsu, Shandong, Guangdong, Zhejiang, Fujian, Hainan, and Liaoning; the central region includes Jilin, Shanxi, Anhui, Hubei, Hunan, Henan, Jiangxi, and Heilongjiang; the western region includes Chongqing, Sichuan, Shaanxi, Guizhou, Yunnan, Gansu, Qinghai, Tibet Autonomous Region, Ningxia Hui Autonomous Region, Xinjiang Uygur Autonomous Region, Guangxi Zhuang Autonomous Region, and Inner Mongolia Autonomous Region.

#### 2.3.3 Pilot survey

The pilot survey was conducted from March to May 2022 among 12 clinical pharmacists and 12 physicians from different hospitals in Nanjing, Jiangsu province. The results showed that the Cronbach *a* reliability coefficient of the clinical pharmacist version PPCI scale was 0.95 and the physician version was 0.98 (the reliability coefficients of the other scales involved in the questionnaire of influencing factors are shown in Tables 2, 3 in Supplementary Material S3). The final survey questionnaire with both the clinical pharmacist version and physician version was finally formed (see Supplementary Material S4), consisting of basic information of the respondents, the PPCI scale to measure the collaboration level, and the questionnaire to measure the influencing factors.

**TABLE 2:** Basic information of sample clinical pharmacists and physicians.

Category	Clinical pharmacist	Physician
	N (%)/mean ± SD^3^	N (%)/mean ± SD
Gender		
Male	198 (41.77)	264 (53.23)
Female	276 (58.23)	232 (46.77)
Age	38.85 ± 7.30	42.13 ± 7.71
Years in practice	11.01 ± 6.81	15.16 ± 7.64
Professional title		
Junior title	201 (42.41)	74 (14.92)
Intermediate title	159 (33.54)	227 (45.77)
Associate senior title	76 (16.03)	125 (25.20)
Senior title	38 (8.02)	70 (14.11)
Terminal degree		
Junior college degree or below	21 (4.43)	7 (1.41)
Bachelor degree	226 (47.68)	198 (39.92)
Master degree	198 (41.77)	229 (46.17)
Doctor degree or above	29 (6.12)	62 (12.50)
Type of employment		
Regular employee	375 (79.11)	421 (84.88)
Non-regular employee	99 (20.89)	75 (15.12)
Area of practice^a^		
General department	152 (32.07)	43 (8.67)
Internal medicine department	180 (37.97)	135 (27.22)
Surgery department	56 (11.81)	78 (15.73)
Gynecology department	36 (7.59)	45 (9.07)
Pediatrics department	50 (10.55)	49 (9.88)
Emergency and critical care departments^1^	72 (15.19)	67 (13.51)
Others^2^	107 (22.57)	116 (23.39)

Note: ^1^Emergency and critical care departments include emergency medicine department, respiratory and critical care medicine department, infection department, and oncology department; ^2^ Others include ophthalmology department, otolaryngology department, stomatology department, dermatology department, anesthesiology department, rehabilitation medicine department, traditional Chinese medicine department, geriatrics department, psychiatry department , pain treatment department, and intervention department; ^3^SD, standard deviation.

^a^ Since a clinical pharmacist/physician may provide clinical services in more than one department, the percentage of the “area of practice” adds up to more than 100%.

**TABLE 3:** Current level of collaboration perceived by clinical pharmacists and physicians.

	Clinical pharmacist					Physician				
	*p*-value of ANOVA	Total score (SD)	Trustworthiness (SD)	Role specification (SD)	Relationship initiation (SD)	*p*-value of ANOVA	Total score (SD)	Trustworthiness (SD)	Role specification (SD)	Relationship initiation (SD)
Total sample		86.84 (10.81)	38.11 (4.79)	29.86 (4.82)	18.87 (2.47)		86.19 (11.81)	37.74 (5.05)	30.02 (4.83)	18.42 (2.72)
Gender	0.835					0.640				
Male		86.72 (11.89)	37.83 (5.47)	29.93 (5.18)	18.95 (2.60)		86.42 (12.10)	37.86 (5.00)	30.12 (4.95)	18.45 (2.85)
Female		86.93 (10.02)	38.30 (4.25)	29.81 (4.56)	18.81 (2.38)		85.92 (11.52)	37.61 (5.12)	29.92 (4.70)	18.39 (2.58)
Age (years)	0.236					0.543				
≤25		83.91 (12.66)	37.45 (4.76)	27.82 (6.35)	18.64 (2.80)		94.17 (3.76)	41.83 (0.41)	31.83 (3.92)	20.50 (0.84)
>25 to ≤35		86.84 (11.84)	37.97 (5.69)	29.92 (5.15)	18.96 (2.59)		85.26 (11.24)	37.13 (4.79)	29.88 (4.57)	18.24 (2.53)
>35 to ≤45		86.18 (11.16)	37.88 (4.64)	29.50 (5.00)	18.80 (2.57)		86.18 (11.66)	37.61 (5.18)	30.12 (4.70)	18.45 (2.57)
>45 to ≤55		88.47 (7.01)	38.81 (3.12)	30.82 (3.13)	18.84 (1.95)		86.07 (12.86)	37.93 (5.18)	29.81 (5.34)	18.33 (3.13)
>55		91.90 (6.33)	40.70 (1.95)	31.60 (3.41)	19.60 (1.78)		88.59 (10.24)	39.36 (3.81)	30.41 (4.36)	18.82 (2.59)
Years in practice	0.589					0.970				
≤5		86.80 (11.81)	38.25 (4.72)	29.60 (5.45)	18.95 (2.65)		85.74 (12.51)	37.54 (5.27)	29.83 (4.99)	18.37 (2.82)
>5 to ≤10		86.89 (10.14)	38.12 (5.00)	29.79 (4.62)	18.99 (2.22)		85.55 (9.43)	37.36 (4.21)	29.94 (3.85)	18.25 (2.25)
>10 to ≤15		86.87 (11.29)	37.81 (4.76)	30.23 (4.71)	18.82 (2.75)		88.31 (10.57)	38.33 (4.71)	31.10 (4.21)	18.88 (2.34)
>15 to ≤20		85.98 (8.34)	38.02 (3.75)	29.33 (4.24)	18.64 (1.80)		84.86 (13.01)	37.32 (5.47)	29.44 (5.28)	18.11 (2.96)
>20		87.80 (13.16)	38.45 (5.57)	30.73 (5.07)	18.61 (3.15)		86.42 (13.34)	38.11 (5.57)	29.79 (5.58)	18.52 (3.16)
Location	0.948					0.006***				
Eastern region		86.97 (9.10)	38.15 (3.88)	29.95 (4.35)	18.87 (2.15)	(Base)	84.73 (13.22)	37.14 (5.58)	29.52 (5.22)	18.08 (3.05)
Central region		86.92 (12.99)	38.12 (6.09)	29.92 (5.48)	18.88 (2.97)	0.001***	88.97 (9.84)	38.76 (3.98)	31.24 (4.21)	18.97 (2.46)
Western region		86.60 (11.11)	38.04 (4.77)	29.70 (4.90)	18.86 (2.46)	0.338	85.92 (10.9)	37.75 (4.96)	29.73 (4.59)	18.45 (2.36)
Grade of hospital	0.142					0.265				
Secondary hospital		87.68 (8.80)	38.12 (4.33)	30.47 (4,18)	19.09 (2.05)		86.85 (11.22)	37.96 (4.72)	30.32 (4.54)	18.57 (2.63)
Tertiary hospital		86.21 (12.10)	38.10 (5.13)	29.41 (5.22)	18.70 (2.74)		85.66 (12.28)	37.57 (5.30)	29.79 (5.05)	18.30 (2.79)
Type of hospital	0.782					0.292				
General hospital		87.18 (9.56)	38.40 (4.10)	29.88 (4.76)	18.91 (2.49)		86.41 (11.04)	37.78 (4.77)	30.13 (4.55)	18.50 (2.52)
Specialized hospital		86.78 (11.02)	38.06 (4.90)	29.86 (4.84)	18.87 (2.48)		84.76 (16.09)	37.50 (6.64)	29.32 (6.39)	17.94 (3.76)
Professional title	0.237					0.048**				
Junior title		85.92 (10.76)	37.65 (5.14)	29.41 (4.92)	18.86 (2.39)	(Base)	83.32 (11.19)	36.51 (4.80)	28.84 (4.62)	17.97 (2.65)
Intermediate title		86.87 (11.61)	38.18 (4.92)	30.01 (5.03)	18.67 (2.68)	0.107	85.87 (12.25)	37.47 (5.26)	30.04 (4.90)	18.36 (2.79)
Associate senior title		88.03 (10.61)	38.59 (4.27)	30.30 (4.50)	19.13 (2.54)	0.006***	88.06 (10.97)	38.74 (4.55)	30.68 (4.48)	18.65 (2.74)
Senior title		89.24 (7.45)	39.24 (2.88)	30.74 (3.90)	19.26 (1.77)	0.069*	86.90 (12.15)	38.16 (5.22)	30.04 (5.30)	18.70 (2.54)
Terminal degree	0.251					0.433				
Junior college degree or below		84.48 (9.55)	37.43 (4.09)	29.19 (4.12)	17.86 (2.83)		84.00 (12.54)	36.71 (5.06)	29.00 (5.45)	18.29 (2.81)
Bachelor degree		86.16 (12.16)	37.90 (5.20)	29.52 (5.40)	18.75 (2.71)		85.83 (12.80)	37.60 (5.58)	29.92 (5.16)	18.32 (2.98)
Master degree		87.51 (9.52)	38.82 (4.59)	30.15 (4.26)	19.04 (2.20)		85.95 (11.44)	37.62 (4.87)	29.95 (4.63)	18.38 (2.63)
Doctor degree or above		89.28 (8.43)	38.79 (3.24)	31.03 (4.03)	19.44 (1.80)		88.44 (9.71)	38.77 (3.77)	30.74 (4.45)	18.92 (2.14)
Type of employment	0.037**					0.618				
Regular employee	0.037**	87.37 (10.60)	38.26 (4.83)	30.13 (4.64)	18.99 (2.42)		86.30 (12.13)	37.78 (5.20)	30.07 (4.89)	18.44 (2.79)
Non-regular employee	(Base)	84.82 (11.47)	37.53 (4.67)	28.86 (5.38)	18.43 (2.63)		85.56 (9.99)	37.51 (4.16)	29.75 (4.54)	18.31 (2.31)

Note: *,**,*** indicate *p* < 0.1, *p* < 0.05, *p* < 0.01, respectively. Variables with *p* > 0.05 are bolded to facilitate reading.

### 2.4 Data analysis

All the original research data were exported from the online questionnaire filling system, and data cleaning and analysis were carried out. Data cleaning was mainly to screen and match valid questionnaires. The exclusion criteria for invalid questionnaires: a) were respondents who did not meet the study requirements, such as those who are not the clinical pharmacists/physicians who are directly providing the clinical treatment or not in the secondary and tertiary hospitals; b) questionnaires that were incomplete; and c) answers that were unrealistic or inconsistent. The matching criteria for valid questionnaires were that with the help of logic functions in Microsoft Excel, the clinical pharmacist version questionnaires and physician version questionnaires from the same hospital could be considered as pairs if the information of investigator number, region, hospital grade, and hospital type were consistent. Meanwhile, if a questionnaire failed to be matched, it was excluded even if valid.

Stata was used to conduct data analysis. First, the filling results of both the versions of the questionnaires were analyzed by using descriptive statistics, which included PPCI scores and current situation of each influencing factor. Before statistical analysis, the normality of each piece of data was tested to understand its distribution, using skewness, kurtosis, Shapiro–Wilk test, and quantile–quantile graphs comprehensively. Also, the reliability of the scales was tested again based on the Cronbach *a* reliability coefficient. The correlation and collinearity between the measured continuous variables in the questionnaire on the influencing factors and the dependent variable PPCI score were examined by using the Pearson correlation coefficient and variance inflation factor (VIF), respectively. Then, the one-way analysis of variance was used to understand the significance of the impact of the interviewees’ sociodemographic information subgroups on their level of cooperation, such as age, years of practice, professional title, educational degree, and so on. Finally, multivariable linear regressions were performed to understand the association between the collaboration level and each influencing factor under the different subjects, respectively, according to the standardized coefficient and significance (*p* < 0.05).

Robustness and heterogeneity tests were also conducted. Among which, the robustness test was performed by changing the regression method, sample size, and dependent variable: a) multiple stepwise regression was used to explore the main factors affecting collaboration, and the *p*-value of removing the variable was set to 0.1 and that of including the variable was set as 0.05; b) winsorizing was conducted for 1% and 99% quartiles of continuous variables in the sample data to confirm that no extreme outliers had a significant effect on the regression results; c) the scores of the three dimensions of PPCI were taken as the dependent variables to test the stability of the basic regression results. Heterogeneity was analyzed on the differences in the influencing factors of the collaboration in the hospitals of different grades, considering the objective gap in the infrastructure, technical strength, and construction of the pharmaceutical department between the secondary and tertiary hospitals.

## 3 Results

### 3.1 Respondents’ information

A total of 960 clinical pharmacists and 953 physicians participated in this study. After screening, there were 536 (55.8%) valid clinical pharmacist version questionnaires and 657 (68.9%) valid physician version questionnaires. After matching, 474 clinical pharmacist version questionnaires and 496 physician version questionnaires were finally involved in data analysis. A total of 281 hospitals from 31 provinces (autonomous regions and municipalities directly under the central government) were included. The distribution of sample hospitals is shown in Table 1. It is basically consistent with the data published in the *China Health Statistical Yearbook 2021*, that is, the research samples can be regarded as representative of the whole.

The basic information of clinical pharmacists and physicians is shown in Table 2. Among them, most (58.23%) of the 474 clinical pharmacists were female, while most (53.23%) of the 496 physicians were male. In terms of age, years of practice, professional title, degree, and the proportion of regular employees in officially budgeted posts, the physicians were slightly higher than the clinical pharmacists. Clinical pharmacists (37.97%) and physicians (27.22%) mainly worked in the internal medicine department.

The results of the full descriptive statistics of the survey are listed in Supplementary Material S2, which include complete information of the respondents, scores of the PPCI, and overall scores of the items and scales in the collaboration factors questionnaire.

### 3.2 Current level of collaboration

Table 3 shows the current level of collaboration between clinical pharmacists and physicians in hospitals in China measured by the PPCI. The average score of the clinical pharmacists was 86.84 (total score, 98), and the average score of the physicians was 86.19 (total score, 98). There was no significant difference in the perceived collaboration level between clinical pharmacists and physicians, indicating that the data in this study can reflect the collaboration progress from the perspectives of both sides objectively and comprehensively to a certain extent.

The results of the one-way analysis of variance showed that the perceived collaboration levels of the pharmacists in officially regular posts, physicians in the central region, and physicians with associate senior title were significantly higher than their base cases (*p* < 0.05). Besides, in terms of the type of hospital, grade of hospital, and educational degree, the general trend was about the same, that is, the clinical pharmacists/physicians perceiving higher levels of collaboration were with higher degrees and in comprehensive hospitals with lower grades; in terms of gender, the perceived collaboration level was slightly higher among female clinical pharmacists and higher among male physicians. There was no obvious linear relationship between age and years in practice with the collaboration level.

### 3.3 Multiple linear regression analysis

The results of normality, reliability, correlation, and multicollinearity tests before regression (see Tables 1–3 in Supplementary Material S3) showed that the variables involved in the statistical analysis could be normally distributed when comprehensively considering the sample size, Q-Q graph, skewness, kurtosis, and other indicators. Also, there was good reliability of the questionnaire, significant correlation between the independent and dependent variables, and no serious collinearity among the variables.

The regression results of both clinical pharmacist data and physician data are shown in Table 4, and the coefficient of determination *R* ^2^ of the clinical pharmacist model was 0.410 while that of the physician was 0.487.

**TABLE 4:** Multiple linear regression results of clinical pharmacist–physician collaboration influencing factors.

Variable	Pharmacist				Physician			
	Coef. (SE)	95% CI	*t*-test (*p*-value)	Standardized Coef	Coef. (SE)	95% CI	*t*-test (*p*-value)	Standardized Coef
1-1 Gender (base: female)								
Male	0.088 (0.945)	(−1.771, 1.946)	0.09 (0.926)	0.004	−0.030 (0.922)	(−1.842, 1.782)	−0.03 (0.974)	−0.001
1-2 Age (years)	0.002 (0.115)	(−0.223, 0.228)	0.02 (0.984)	0.002	0.043 (0.144)	(−0.240, 0.325)	0.3 (0.767)	0.028
1-3 Years in practice	−0.113 (0.118)	(−0.344, 0.118)	−0.96 (0.338)	−0.071	−0.066 (0.143)	(−0.346, 0.215)	−0.46 (0.647)	−0.042
1-4 Location (base: eastern region)								
Central region	−1.323 (1.187)	(−3.656, 1.009)	−1.12 (0.265)	−0.053	2.284 (1.144)	(0.036, 4.532)	2 (0.046**)	**0.084**
Western region	0.062 (1.106)	(−2.113, 2.237)	0.06 (0.955)	0.003	0.208 (1.064)	(−1.884, 2.300)	0.2 (0.845)	0.008
1-5 Area of practice								
General department	−1.143 (1.413)	(−3.921, 1.636)	−0.81 (0.419)	−0.049	−1.602 (2.165)	(−5.856, 2.652)	−0.74 (0.460)	−0.038
Internal medicine department	−0.997 (1.03)	(−3.022, 1.029)	−0.97 (0.334)	−0.045	−0.515 (1.698)	(−3.853, 2.822)	−0.3 (0.762)	−0.019
Surgery department	0.521 (1.398)	(−2.227, 3.269)	0.37 (0.710)	0.016	−0.229 (1.786)	(−3.739, 3.281)	−0.13 (0.898)	−0.007
Gynecology department	2.812 (1.749)	(−0.627, 6.251)	1.61 (0.109)	0.069	−2.933 (2.184)	(−7.226, 1.359)	−1.34 (0.180)	−0.071
Pediatrics department	−1.581 (1.510)	(−4.550, 1.388)	−1.05 (0.296)	−0.045	−2.81 (1.994)	(−6.729, 1.109)	−1.41 (0.160)	−0.071
Emergency and critical care department	−0.080 (1.299)	(−2.633, 2.472)	−0.06 (0.951)	−0.003	−0.819 (1.681)	(−4.123, 2.485)	−0.49 (0.626)	−0.024
Others	0.811 (1.134)	(−1.417, 3.039)	0.72 (0.475)	0.031	0.487 (1.726)	(−2.905, 3.879)	0.28 (0.778)	0.017
1-6 Type of hospital (base: specialized hospital)								
General hospital	0.412 (1.319)	(−2.181, 3.005)	0.31 (0.755)	0.013	2.046 (1.350)	(−0.608, 4.700)	1.52 (0.130)	0.059
1-7-1 Grade of hospital (base: secondary hospital)								
Tertiary hospital	−1.644 (1.011)	(−3.630,0.343)	−1.63 (0.105)	−0.075	−2.199 (0.933)	(−4.034, −0.365)	−2.36 (0.019**)	**−0.092**
1-7-2 Clinical pharmacist training base (base: no)	−0.287 (1.062)	(−2.375, 1.801)	−0.27 (0.787)	−0.013				
1-8-1 Professional title (base: junior title)								
Intermediate title	−0.341 (1.095)	(−2.492, 1.811)	−0.31 (0.756)	−0.015	1.460 (1.411)	(−1.312, 4.233)	1.04 (0.301)	0.062
Associate senior title	0.129 (1.508)	(−2.836, 3.094)	0.090 (0.932)	0.004	2.433 (1.651)	(−0.812, 5.678)	1.47 (0.141)	0.089
Senior title	0.030 (2.041)	(−3.982, 4.043)	0.01 (0.988)	0.001	1.881 (1.974)	(−1.999, 5.760)	0.95 (0.341)	0.055
1-8-2 Type of employment (base: non-regular employee)								
Regular employee	0.200 (1.142)	(−2.044, 2.445)	0.18 (0.861)	0.008	−2.709 (1.282)	(−5.230, −0.188)	−2.11 (0.035**)	**−0.082**
1-8-3 Type of practice								
Specialized clinical pharmacist (base: general)	−0.066 (1.280)	(−2.583, 2.450)	−0.05 (0.959)	−0.003	−1.292 (0.949)	(−3.157, 0.573)	−1.36 (0.174)	−0.054
Full-time clinical pharmacist (base: part-time)	2.528 (1.796)	(−1.003, 6.059)	1.41 (0.160)	0.066	−1.440 (1.876)	(−5.127, 2.248)	−0.77 (0.443)	−0.029
1-9-1 Terminal degree (base: junior college degree or below)								
Bachelor degree	−2.502 (2.305)	(−7.033, 2.029)	−1.09 (0.278)	−0.116	5.068 (3.634)	(−2.075, 12.211)	1.39 (0.164)	0.21
Master degree	−1.458 (2.353)	(−6.084, 3.168)	−0.62 (0.536)	−0.066	6.052 (3.688)	(−1.196, 13.300)	1.64 (0.101)	0.255
Doctor degree or above	0.18 (3.024)	(−5.764, 6.123)	0.06 (0.953)	0.004	7.739 (3.880)	(0.112, 15.366)	1.99 (0.047**)	**0.217**
1-9-2 Major (base: clinical pharmacy)								
Other pharmacy-allied majors	1.272 (1.089)	(−0.868, 3.413)	1.17 (0.243)	0.056				
Non-pharmaceutical-related majors	1.474 (2.345)	(−3.136, 6.085)	0.63 (0.53)	0.029				
1-9-3 Pattern of training (base: training after graduation)								
Training after job transfer	0.035 (1.131)	(−2.187, 2.257)	0.03 (0.975)	0.002				
Direct assignment without training	−9.733 (3.425)	(−16.466, −3.000)	−2.84(0.005***)	**−0.129**				
1-10-1 Interdisciplinary education experience								
Have taken basic medical/pharmaceutical courses (base: no)	−1.790 (1.095)	(−3.941, 0.362)	−1.63 (0.103)	−0.072	−1.691 (1.248)	(−4.145, 0.763)	−1.35 (0.176)	−0.054
Have taken management science courses (base: no)	1.314 (1.268)	(−1.178, 3.806)	1.04 (0.300)	0.043	−0.015 (1.132)	(−2.240, 2.210)	−0.01 (0.989)	−0.001
1-10-2 Inter-career working experience (base: no)	1.832 (1.302)	(−0.728, 4.392)	1.41 (0.160)	0.065	0.157 (1.489)	(−2.769, 3.083)	0.11 (0.916)	0.004
1-10-3 Interprofessional collaboration experience	0.032 (0.269)	(−0.498, 0.562)	0.12 (0.906)	0.006	−0.145 (0.258)	(−0.651, 0.361)	−0.56 (0.574)	−0.022
1-11 Personality								
1-11-1 Extraversion	0.006 (0.503)	(−0.984, 0.995)	0.01 (0.991)	0.001	0.604 (0.515)	(−0.407, 1.615)	1.17 (0.241)	0.051
1-11-2 Agreeableness	0.687 (0.687)	(−0.663, 2.038)	1 (0.318)	0.046	0.191 (0.595)	(−0.979, 1.361)	0.32 (0.749)	0.012
1-11-3 Conscientiousness	2.316 (0.836)	(0.673, 3.959)	2.77 (0.006***)	**0.141**	−1.57 (0.776)	(−3.095, −0.044)	−2.02 (0.044**)	**−0.089**
1-11-4 Neuroticism	−0.29 (0.694)	(−1.655, 1.075)	−0.42 (0.676)	−0.022	0.346 (0.700)	(−1.029, 1.721)	0.49 (0.621)	0.023
1-11-5 Openness	0.806 (0.566)	(−.306, 1.918)	1.42 (0.155)	0.061	0.297 (0.575)	(−0.834, 1.427)	0.52 (0.606)	0.02
2-1 Institution administration								
2-1-1 System construction	0.878 (1.039)	(−1.163, 2.920)	0.85 (0.398)	0.053	5.040 (1.318)	(2.449, 7.630)	3.82 (0.000***)	**0.233**
2-1-2 Manager support	2.658 (1.112)	(0.472, 4.845)	2.39 (0.017**)	**0.172**	2.717 (1.087)	(0.580, 4.853)	2.5 (0.013**)	**0.151**
2-1-3 Personnel development	−0.222 (1.242)	(−2.663, 2.219)	−0.18 (0.858)	−0.013	0.497 (1.254)	(−1.967, 2.962)	0.40 (0.692)	0.023
2-2 Job satisfaction								
2-2-1 Workload	0.534 (0.592)	(−0.630, 1.699)	0.90 (0.368)	0.045	−0.070 (0.554)	(−1.159, 1.019)	−0.13 (0.900)	−0.006
2-2-2 Working environment	0.867 (0.776)	(−0.659, 2.393)	1.12 (0.265)	0.053	0.625 (0.808)	(−0.963, 2.212)	0.77 (0.440)	0.036
2-2-3 Remuneration	0.896 (0.745)	(−0.569, 2.361)	1.20 (0.230)	0.071	1.192 (0.735)	(−0.253, 2.638)	1.62 (0.106)	0.085
2-3 Working atmosphere								
2-3-1 Team acceptability	0.159 (0.111)	(−0.059, 0.378)	1.43 (0.153)	0.063	−0.055 (0.113)	(−0.277, 0.167)	−0.48 (0.629)	−0.02
2-3-2 Social acceptability	0.732 (0.979)	(−1.191, 2.656)	0.75 (0.455)	0.042	−0.954 (1.378)	(−3.662, 1.755)	−0.69 (0.489)	−0.041
2-4 Resources and conditions								
2-4-1 Adequate personnel allocation (base: no)	−1.102 (0.985)	(−3.037, 0.834)	−1.12 (0.264)	−0.051	−0.637 (0.913)	(−2.433, 1.158)	−0.70 (0.486)	−0.027
2-4-2 Number of required facilities and equipment	0.047 (0.148)	(−0.245, 0.339)	0.32 (0.752)	0.015	−0.222 (0.254)	(−0.721, 0.277)	−0.87 (0.382)	−0.035
2-5 Collaboration incentives (base: no)	−2.081 (1.042)	(−4.130, −0.032)	−2.00 (0.047**)	**−0.094**	−0.035 (0.993)	(−1.988, 1.917)	−0.04 (0.972)	−0.001
3-1 Respect	2.186 (1.264)	(−0.298, 4.670)	1.73 (0.084*)	0.131	−2.489 (1.506)	(−5.45, 0.471)	−1.65 (0.099*)	−0.11
3-2 Trust	0.453 (1.144)	(−1.795, 2.702)	0.4 (0.692)	0.024	3.675 (1.329)	(1.062, 6.288)	2.76 (0.006***)	**0.169**
3-3 Role recognition	−0.099 (1.287)	(−2.629, 2.432)	−0.08 (0.939)	−0.004	0.564 (1.279)	(−1.95, −3.078)	0.44 (0.659)	0.024
3-4 Fairness	−1.372 (1.058)	(−3.452, 0.708)	−1.30 (0.195)	−0.082	−2.936 (0.966)	(−4.834, −1.038)	−3.04(0.003***)	**−0.174**
3-5 Attitude to collaboration								
3-5-1 Job burnout	0.133 (0.156)	(−0.175, 0.440)	0.85 (0.397)	0.044	0.041 (0.135)	(−0.224, 0.306)	0.30 (0.762)	0.013
3-5-2 Usefulness perception	0.249 (0.268)	(−0.277, 0.776)	0.93 (0.352)	0.042	0.965 (0.267)	(0.441, 1.489)	3.62 (0.000***)	**0.149**
3-5-3 Shared expectation	0.847 (0.394)	(0.073, 1.621)	2.15 (0.032**)	**0.116**	0.888 (0.306)	(0.287, 1.489)	2.90 (0.004***)	**0.132**
3-6 Leadership								
3-6-1 Power influence (base: same office rank)								
Higher rank of physician	−1.216 (0.971)	(−3.124, 0.693)	−1.25 (0.211)	−0.056	−2.369 (1.020)	(−4.373, −0.364)	−2.32 (0.021**)	**−0.098**
Higher rank of clinical pharmacist	0.709 (1.980)	(−3.184, 4.601)	0.36 (0.721)	0.016	−0.223 (1.574)	(−3.316, 2.87)	−0.14 (0.888)	−0.006
3-6-2 Non-power influence								
Effect of morality and sentiment (base: no)	1.963 (0.950)	(0.095, 3.832)	2.07 (0.039**)	**0.087**	0.782 (0.914)	(−1.013, 2.578)	0.86 (0.392)	0.032
Effect of knowledge and ability (base: no)	1.807 (1.927)	(−1.981, 5.596)	0.94 (0.349)	0.039	−0.665 (2.400)	(−5.382, 4.051)	−0.28 (0.782)	−0.011
3-7 Familiarity								
3-7-1 Member mobility	−0.444 (0.650)	(−1.722, 0.834)	−0.68 (0.495)	−0.032	0.039 (0.609)	(−1.158, 1.235)	0.06 (0.950)	0.003
3-7-2 Out-of-work socializing	−0.276 (0.580)	(−1.416, 0.865)	−0.48 (0.635)	−0.023	0.122 (0.546)	(−0.951, 1.196)	0.22 (0.823)	0.01
3-8 Communication								
3-8-1 Team communication ability	0.981 (1.396)	(−1.764, 3.726)	0.70 (0.483)	0.048	0.842 (1.360)	(−1.83, 3.514)	0.62 (0.536)	0.041
3-8-2 General language ability	3.188 (1.328)	(0.578, 5.799)	2.40 (0.017**)	**0.155**	3.664 (1.402)	(0.908, 6.419)	2.61 (0.009***)	**0.176**
Constant	10.189 (9.299)	(−8.092,28.469)	1.10 (0.274)	-	19.061 (9.517)	(0.356, 37.766)	2.00 (0.046)	-
*SR* ^2^	0.410				0.487			
Adjusted *R* ^2^	0.320				0.419			

^a^ *,**,*** indicate *p* < 0.1, *p* < 0.05, *p* < 0.01, respectively. Variables with *p* > 0.05 are bolded to facilitate reading.

^b^ Blank parts in the columns of “physician” indicate that this factor is only measured for clinical pharmacists. See Supplement 4 for the complete questionnaire.

According to Table 4, for clinical pharmacists, in terms of participant characteristics, when compared to standardized training after graduation, the pattern that directly assigned a pharmacist (X1-9-3) who is originally responsible for drug dispensing as the clinical pharmacist without training had a significant negative impact on the collaboration (standardized coefficient, hereafter referred to as Coef. = −0.129, *p* < 0.01). Also, the stronger sense of responsibility (X1-11-3) of clinical pharmacists had a significant positive influence on the level of collaboration (Coef. = 0.141, *p* < 0.01). In terms of context characteristics, hospital and department managers supporting the clinical pharmacists’ work and collaboration (X2-1-2) were regarded significant facilitators of collaboration (Coef. = 0.172, *p* < 0.05). However, setting a separate incentive mechanism (X2-5) for pharmaceutical service and collaboration might be a barrier to the collaboration of clinical pharmacists (Coef. = −0.094, *p* < 0.05). In terms of exchange characteristics, clinical pharmacists (X3-8-2) with good communication skills (Coef. = 0.155, *p* < 0.05) (X3-5-3) shared good expectations and beliefs for collaboration (Coef. = 0.116, *p* < 0.05), while those (X3-6-3) attracted by good moral emotions of physicians (Coef. = 0.087, *p* < 0.05) perceived significantly higher levels of collaboration.

For physicians, in terms of participant characteristics, physicians in the central region (X1-4) when compared with those in the eastern region (Coef. = 0.084, *p* < 0.05), and physicians with a doctor’s degree or above (X1-9-1) when compared with those with an associate’s degree or below (Coef. = 0.217, *p* < 0.05) perceived significantly higher levels of collaboration. However, physicians (X1-8-2) in officially regular posts (Coef. = −0.082, *p* < 0.05), those (X1-7-1) in hospitals of a higher grade (Coef. = −0.092, *p* < 0.05), and those (X1-11-3) having a high sense of responsibility (Coef. = −0.089, *p* < 0.05) had negative impacts on the collaboration among physicians. In terms of context characteristics, hospital and department managers (X2-1-1) could develop perfect and clear system documents (Coef. = 0.151, *p* < 0.01) and support collaborative work (X2-1-2) (Coef. = 0.233, *p* < 0.05), that had significantly positive impacts on collaboration. In terms of exchange characteristics, physicians (X3-2) who showed trust toward clinical pharmacists (X3-5-2) (Coef. = 0.169, *p* < 0.01) clearly recognized the value of collaboration (Coef. = 0.149, *p* < 0.01), while those who (X3-5-3) clearly identified the joint responsibility and expectation of the collaboration (Coef. = 0.132, *p* < 0.01) and those (X3-8-2) who enjoyed good communication skills with clinical pharmacists (Coef. = 0.176, *p* < 0.01) perceived a significantly higher level of collaboration. However, physicians (X3-6-1) with clear power disparity (Coef. = −0.098, *p* < 0.05) and with very strong perceptions of fairness (X3-4) in efforts and rewards (Coef. = −0.174, *p* < 0.01) have negative impacts on the collaboration with physicians.

### 3.4 Robustness analysis and heterogeneity analysis

The results of the regression stability test (see Tables 4, 5 in Supplementary Material S3) showed that when compared with the results of basic regression, most significant explanatory variables statistically still had effects on the level of collaboration between clinical pharmacists and physicians, and the positive or negative nature of the coefficient did not change, indicating that the results of basic regression were relatively robust. Among these, the results of multiple stepwise regression showed that team acceptability (X2-3-1) (Coef. = 0.196, *p* < 0.05), remuneration (X2-2-3) (Coef. = 1.291, *p* < 0.05), and respect (X3-1) (Coef. = 2.375, *p* < 0.05) may also have a significant impact on the level of cooperation, while the significance of collaboration incentives, type of employment, and education level disappeared.

**TABLE 5:** Results of heterogeneity analysis.

Variable	Clinical pharmacist		Physician	
	Secondary	Tertiary	Secondary	Tertiary
	Standardized *ß* (*p*-value)	Standardized *ß* (*p*-value)	Standardized *ß* (*p*-value)	Standardized *ß* (*p*-value)
1-1 Gender (base: female)				
Male	0.035 (0.583)	−0.029 (0.639)	−0.073 (0.240)	0.067 (0.218)
1-2 Age	−0.138 (0.239)	−0.035 (0.758)	−0.042 (0.764)	0.026 (0.851)
1-3 Years in practice	0.101 (0.382)	−0.013 (0.904)	0.172 (0.217)	−0.170 (0.209)
1-4 Location (base: eastern region)				
Central region	−0.037 (0.590)	−0.097 (0.160)	0.063 (0.321)	0.141 (0.026**)
Western region	0.060 (0.389)	−0.030 (0.667)	−0.01 (0.872)	0.041 (0.498)
1-5 Area of practice				
General department	0.027 (0.773)	−0.178(0.047**)	0.039 (0.637)	−0.034 (0.643)
Internal medicine department	0.074 (0.287)	−0.073 (0.274)	−0.051 (0.570)	0.103 (0.321)
Surgery department	−0.038 (0.558)	0.034 (0.573)	−0.051 (0.525)	0.086 (0.319)
Gynecology department	0.172(0.010**)	0.021 (0.722)	−0.094 (0.239)	0.003 (0.973)
Pediatrics department	−0.030 (0.663)	−0.08 (0.186)	−0.074 (0.292)	−0.034 (0.682)
Emergency and critical care department	−0.060 (0.327)	0.027 (0.659)	0.039 (0.597)	−0.012 (0.872)
Others	0.008 (0.902)	0.068 (0.278)	0.008 (0.926)	0.109 (0.286)
1-6 Type of hospital (base: specialized hospital)				
General hospital	0.044 (0.488)	−0.074 (0.241)	0.051 (0.424)	0.052 (0.356)
1-7-2 Clinical pharmacist training base (base: no)	−0.013 (0.837)	−0.022 (0.747)		
1-8-1 Professional title (base: junior title)				
Intermediate title	−0.06 (0.415)	0.001 (0.986)	0.043 (0.656)	0.083 (0.328)
Associate senior title	0.08 (0.288)	−0.007 (0.918)	0.008 (0.939)	0.200 (0.022**)
Senior title	−0.063 (0.395)	0.000 (0.995)	−0.015 (0.862)	0.114 (0.204)
1-8-2 Type of employment (base: non-regular employee)				
Regular employee	0.037 (0.564)	−0.039 (0.540)	−0.015 (0.816)	−0.044 (0.46)
1-8-3 Type of practice				
Specialized clinical pharmacist (base: general)	0.000 (0.999)	−0.131 (0.137)	0.034 (0.576)	−0.105 (0.066*)
Full-time clinical pharmacist (base: part-time)	0.016 (0.831)	0.135(0.036**)	0.010 (0.869)	−0.08 (0.143)
1-9-1 Terminal degree (base: junior college degree or below)				
Bachelor degree	0.123 (0.426)	−0.254 (0.125)	−0.030 (0.874)	0.368 (0.187)
Master degree	0.172 (0.249)	−0.143 (0.400)	0.038 (0.838)	0.388 (0.207)
Doctor degree or above	0.107 (0.276)	0.001 (0.995)	0.063 (0.613)	0.311 (0.169)
1-9-2 Major (base: clinical pharmacy)				
Other pharmacy-allied majors	0.027 (0.714)	0.036 (0.588)		
Non–pharmaceutical-related majors	0.044 (0.559)	0.040 (0.508)		
1-9-3 Pattern of training (base: training after graduation)				
Training after job transfer	0.071 (0.356)	−0.044 (0.525)		
Direct assignment without training	0.092 (0.252)	−0.185(0.003***)		
1-10-1 Interdisciplinary education experience				
Have taken basic medical/pharmaceutical courses (base: no)	−0.103 (0.138)	−0.101 (0.105)	−0.015 (0.817)	−0.07 (0.202)
Have taken management science courses (base: no)	0.099 (0.128)	0.016 (0.784)	0.005 (0.931)	−0.030 (0.581)
1-10-2 Inter-career working experience (base: no)	0.050 (0.498)	0.073 (0.247)	−0.013 (0.844)	0.020 (0.714)
1-10-3 Interprofessional collaboration experience	−0.076 (0.306)	0.062 (0.388)	−0.023 (0.722)	0.001 (0.988)
1-11 Personality				
1-11-1 Extraversion	0.088 (0.220)	−0.095 (0.172)	0.035 (0.613)	0.053 (0.387)
1-11-2 Agreeableness	−0.029 (0.668)	0.113 (0.077*)	0.053 (0.372)	−0.032 (0.566)
1-11-3 Conscientiousness	0.090 (0.251)	0.194(0.008***)	−0.104 (0.174)	−0.089 (0.156)
1-11-4 Neuroticism	0.058 (0.468)	−0.021 (0.775)	−0.055 (0.472)	0.066 (0.316)
1-11-5 Openness	0.068 (0.309)	0.050 (0.407)	0.058 (0.373)	−0.043 (0.451)
2-1 Institution administration				
2-1-1 System construction	0.149 (0.126)	−0.007 (0.932)	0.313 (0.004***)	0.161 (0.049**)
2-1-2 Manager support	0.223(0.038**)	0.250(0.019**)	0.042 (0.666)	0.173 (0.043**)
2-1-3 Personnel development	−0.069 (0.541)	−0.071 (0.485)	−0.068 (0.470)	0.121 (0.138)
2-2 Job satisfaction				
2-2-1 Workload	0.048 (0.528)	0.064 (0.373)	−0.077 (0.310)	0.013 (0.829)
2-2-2 Working environment	0.044 (0.510)	0.083 (0.235)	−0.01 (0.896)	0.103 (0.101)
2-2-3 Remuneration	0.054 (0.552)	0.020 (0.810)	0.139 (0.125)	0.113 (0.123)
2-3 Working atmosphere				
2-3-1 Team acceptability	−0.112 (0.132)	0.169(0.005***)	0.085 (0.190)	−0.087 (0.138)
2-3-2 Social acceptability	0.024 (0.781)	−0.024 (0.766)	−0.038 (0.690)	−0.116 (0.174)
2-4 Resources and conditions				
2-4-1 Adequate personnel allocation (base: no)	−0.086 (0.220)	−0.051 (0.434)	−0.083 (0.167)	0.005 (0.924)
2-4-2 Numbers of needed facilities and equipment	0.014 (0.841)	0.022 (0.746)	−0.001 (0.983)	−0.066 (0.236)
2-5 Collaboration incentives (base: no)	0.004 (0.955)	−0.125 (0.062*)	−0.029 (0.663)	0.037 (0.509)
3-1 Respect	0.097 (0.369)	0.150 (0.185)	−0.116 (0.275)	−0.126 (0.178)
3-2 Trust	−0.063 (0.477)	0.102 (0.242)	0.088 (0.344)	0.241 (0.009***)
3-3 Role recognition	0.153 (0.073*)	−0.066 (0.429)	0.135 (0.111)	−0.060 (0.453)
3-4 Fairness	0.007 (0.943)	−0.070 (0.434)	−0.173 (0.043**)	−0.258 (0.004***)
3-5 Attitude to collaboration				
3-5-1 Job burnout	0.065 (0.377)	0.070 (0.358)	0.017 (0.808)	0.012 (0.846)
3-5-2 Usefulness perception	0.040 (0.534)	0.040 (0.534)	0.105 (0.091*)	0.211 (0.001***)
3-5-3 Shared expectation	0.209(0.013**)	0.107 (0.176)	0.178 (0.010**)	0.103 (0.118)
3-6 Leadership				
3-6-1 Power influence (base: same office rank)				
Higher rank of physician	−0.081 (0.223)	−0.024 (0.695)	−0.029 (0.672)	−0.111 (0.061*)
Higher rank of clinical pharmacist	0.005 (0.935)	0.039 (0.534)	0.035 (0.608)	−0.004 (0.947)
3-6-2 Non-power influence				
Effect of morality and sentiment (base: no)	0.094 (0.158)	0.067 (0.261)	0.006 (0.914)	0.006 (0.91)
Effect of knowledge and ability (base: no)	0.045 (0.495)	−0.004 (0.94)	0.059 (0.325)	−0.085 (0.141)
3-7 Familiarity				
3-7-1 Member mobility	−0.071 (0.358)	0.023 (0.729)	0.014 (0.846)	−0.026 (0.684)
3-7-2 Out-of-work socializing	−0.045 (0.543)	−0.019 (0.78)	−0.040 (0.564)	0.036 (0.565)
3-8 Communication				
3-8-1 Team communication ability	0.013 (0.893)	0.116 (0.259)	0.129 (0.176)	0.027 (0.789)
3-8-2 General language ability	0.205 (0.035**)	0.060 (0.516)	0.317 (0.002***)	0.179 (0.077*)

^a^ *,**,*** indicate *p* < 0.1, *p* < 0.05, *p* < 0.0,=1, respectively. Variables with *p* > 0.05 are bolded to facilitate reading.

^b^ Blank parts in the columns of “physician” indicate that this factor is only measured for clinical pharmacists. See Supplement 4 for the complete questionnaire.

The heterogeneity analysis results are shown in Table 5. Clinical pharmacists, due to the more advanced processes of clinical pharmacy construction in tertiary hospitals, pay more attention to factors that support and guarantee systematic and active clinical collaboration, such as the depth to which clinical pharmacists engage in clinical practice, training standardization, work responsibility, and team atmosphere. However, clinical pharmacists in secondary hospitals think highly of the basic quality required to form collaborations, such as institutional management, common expectation, and communication ability. Physicians in tertiary hospitals have higher requirements for professional and technical competence on the other party in a collaboration. They prefer to pay more attention to the trustworthiness of clinical pharmacists, practical value of the collaboration in reducing workload and improving the quality of treatment, and attitude of managers. Those in secondary hospitals, however, tend to consider interaction factors such as mutual communication and information sharing as well as shared responsibilities and expectations.

## 4 Discussion

### 4.1 Significant factors influencing the collaboration between clinical pharmacists and physicians

According to the multiple linear regression results, manager support (X2-1-2), shared expectation (X3-5-3), and general language communication ability (X3-8-2) all passed the 5% significance test in both the clinical pharmacist and physician groups, and the coefficient was positive. This result is consistent with the findings of existing studies (Annelies et al., 2018; Williams et al., 2018; Dähne et al., 2019) that it will be conducive to the collaboration between clinical pharmacists and physicians if managers support clinical pharmacists to go deep into the clinic by reducing the basic drug management work and ensuring their independence and right to participate in decision-making; if hospitals can provide the available facilities and equipment for patient information exchange and skill training to improve face-to-face communication quality; and if clinical pharmacists and physicians can maintain a common belief and responsibility to improve patient service by collaboration.

In addition, standardized training (X1-9-3), team acceptability (X2-3-1), and non-power influence (X3-6-2) passed the significance test at the 5% level in the clinical pharmacist group, while degree (X1-9-1), system construction (X2-1-1), remuneration (X2-2-3), trust (X3-2), perception of usefulness (X3-5-2), and power influence (X3-6-1) passed the significance test at 5% level in the physician group. The results are basically consistent with the findings of previous studies (Tan et al., 2013; Weissenborn et al., 2017; Alhossan and Alazba, 2019; Hatton et al., 2021). Obtaining standardized training qualification is the foundation for clinical pharmacists to begin practice. Through full-time learning and practice in the training bases, clinical pharmacists can enrich clinical drug knowledge, improve medication practice and clinical communication skills, and boost the ability of medical records writing and literature review to help clinical pharmacists gain the trust of physicians and win a better collaboration. Meanwhile, issuing clear regulations or guidelines by hospitals or official regulatory agencies can not only define the scope of authority and responsibility and form a standard work model to ensure the stability and compliance of collaboration but also help physicians to have a more comprehensive cognition of the clinical value of collaborations, such as reducing the physicians’ workload, improving the quality of treatment, and reducing unnecessary drug costs, so as to improve their enthusiasm and inclusiveness of integration. Moreover, both power and non-power influence of collaborators will also exert effects on collaboration. Power influence means that one party reasonably and properly takes advantage of their position or seniority to instruct the other party to execute instructions correctly. If the power gap between the two parties is too large, however, the negative psychology of hierarchy oppression may be generated and change the positive attitude toward collaboration. Non-power influence is caused by the leader’s relative good competence and moral cultivation, and mainly includes morality, talent, knowledge, and emotion, which are the motivation for natural and positive behavior of the other members in the team and often has a stronger and more lasting impact, contributing to a better collaboration.

There were still some factors that passed the significance test, but their influencing mechanisms are up for debate:

First, higher levels of collaboration reported by non-regular physicians (X1-8-2) in the central region (X1-4) and secondary hospitals (X1-7-1) were comparable to those in the eastern region and tertiary hospitals where the level of economy and healthcare is higher. This may be because physicians located in these regions and hospitals with a relatively lower level of clinical pharmacy construction may have lower expectations on clinical pharmacists’ professional ability and their value in collaboration. They may believe that it is enough for clinical pharmacists to provide just the basic drug information and assistance in drug monitoring, with no high requirements on their participation in drug decision-making and specialist consultation, and so they tend to have a perceived higher level of collaboration.

Second, a stronger sense of conscientiousness (X1-11-3) was associated with a higher level of collaboration in the clinical pharmacist group, but the opposite was true in the physician group. The sense of conscientiousness is one of personality characteristics, driving people to achieve perfection and perseverance. For clinical pharmacists, as the active party in integration, they should take the initiative to improve professional skills to meet clinical needs. Therefore, clinical pharmacists with a stronger sense of responsibility can have a stronger sense of achievement and report a higher level of collaboration. While for physicians, as the receiving end of integration, the stronger the sense of responsibility, the higher their expected value of clinical pharmacists and collaborative relationship, so less-than-stellar collaborative experiences can lower their perceived collaboration level.

Third, the perceived level of collaboration among clinical pharmacists who are given incentives (X2-5) was lower. In international studies on pharmacists, financial compensation has always been the focus of discussion. It has been acknowledged that pharmacists in institutions that lack a clear compensation mechanism for professional pharmaceutical services often lack the motivation to provide more high-quality services. However, the actual impact of separate charge for clinical pharmacy or collaboration service in China has to be considered in multiple dimensions. For example, in the case where relevant laws and regulations on pharmaceutical care charge have not been established, there would be problems such as illegal repeating charges by disaggregating hospitalization processes, large ability gaps among clinical pharmacists, and lower social recognition, which hinder the maintenance of normal medical treatment order and collaborative relationship.

Finally, the greater the physicians' perceived fairness (X3-4) of efforts and rewards, the lower their reported level of collaboration. This may be because they tend to believe that the output of their work has reached saturation with regard to the requirements of their department and, in this case, may lack the motivation to further collaborate to improve treatment results with such high job satisfaction.

### 4.2 Suggestions for promoting clinical pharmacists’ integration into multidisciplinary teams

At the national level, unified and clear norms on clinical pharmacists’ work and collaboration should be issued as soon as possible, to formulate the legal framework of work objectives, role specification, and division of powers and responsibilities; improve the quality and standardization; and avoid the fragmentation of patient care. It is an important institutional guarantee for clinical pharmacists to carry out their work and form effective collaboration.

As to the cultivation of clinical pharmacists' talents at the stage of college education and on-the-job training, in countries where clinical pharmacy has not been fully developed, attention should be paid to adjust the educational mode of clinical pharmacists, raising the occupational access threshold, improving standardized training, and cultivating problem-solving clinical ability, thus ensuring the professional quality and comprehensive quality of clinical pharmacists. In the United States, the American Association of Pharmaceutical Colleges (ACCP) set the Doctor of Pharmacy as the entry threshold for licensed pharmacists, and those who have obtained the degree can take the post of resident pharmacist and receive standardized training sponsored by the American Academy of Hospital Pharmacists (ASHP). The training program includes two phases: general training during Postgraduate Year One (PGY-1) through rotations in various departments and specialized training in PGY-2 by focusing on analyzing complex cases. In addition, attention should be paid to the cultivation of humanistic literacy, and training sessions on organizational behavior, psychology, and communication skills should be set up regularly. ACCP believes that communication ability is one of the competencies necessary for clinical pharmacy practitioners (Saseen et al., 2017), that is, to communicate effectively with patients, guardians, or other healthcare professionals and stakeholders on patients’ therapy information and provide concise advice, maintaining confidence, compassion, and respect.

### 4.3 Limitations

First, the survey methodology could have introduced selection biases. The sampling methods where clinical pharmacists with collaborative experience recommend familiar physicians could result in hospitals without clinical pharmacists or collaborative working mode being excluded and with a relatively higher score of collaboration. Second, some scales in the questionnaire have not been verified in the Chinese population or among clinical pharmacists/physicians, although several expert consultations and pilot surveys have been conducted to test their quality. Finally, the research results and discussion require further expert argumentation to demonstrate their actual function and applicability in real collaboration practice.

## 5 Conclusion

Taking the interprofessional collaboration between clinical pharmacists and physicians as the focus, this study aimed to analyze the relevant influencing factors of clinical pharmacists’ integration into the multidisciplinary team of clinical drug therapy. Since comprehensively sorting out the possible influencing factors, a national survey was conducted in secondary and tertiary hospitals in China to analyze the actual influencing mechanism of each factor under three dimensions of participant, context, and exchange characteristics. It is the first empirical study in this field in China and could act as a baseline data set for analyzing the current situation of collaboration in China and other countries with similar health systems and providing reference for health policymakers.

## Data Availability

The original contributions presented in the study are included in the article/Supplementary Material, further inquiries can be directed to the corresponding authors.
