# Supplementary material for: Factors influencing clinical pharmacists’ integration into the clinical multidisciplinary care team
Source: Front Pharmacol. 2023 Jun 12;14:1202433. doi: 10.3389/fphar.2023.1202433 (PMC10291231; doi:10.3389/fphar.2023.1202433)
Supplement: Supplementary file 1 [file Table1.docx]

Supplementary Material

**Factors Influencing Clinical Pharmacists’ Integration into the Clinical Multidisciplinary Care Team**

Chenyu WANG^1^, Maozhen LI^1^, Yuankai HUANG^1*^, Xiaoyu XI^1*^

**^*^ Corresponding author**

Dr. Xiaoyu XI E-mail: [xixy@cpu.edu.cn](mailto:xixy@cpu.edu.cn).

Dr. Yuankai HUANG E-mail: [hyk@cpu.edu.cn](mailto:hyk@cpu.edu.cn).

**Supplement 1 Selection of variables and measurement instruments**

The theoretical basis of this study is to systematically sort out the factors that may affect the collaboration between clinical pharmacists and physicians. Therefore, existing literature, published before April 2022 on Web of science, PubMed and other databases and focusing on relevant keywords, such as “pharm*”, “physician OR doctor OR general practitioner”, “collaborat* OR cooperat* OR inter-profession* OR multidisciplin* OR co-work”, “influencing factor OR impact OR facilitat* OR barrier”, were searched and screened. Finally, 26 qualitative or quantitative studies that meet the research needs are included to extract the influencing factors and corresponding mechanisms reported in them.

Due to the differences in the classification standard and expression of influencing factors in different studies, there are still some problems of parallel or overlapping connotations among those influencing factors directly extracted above. In this regard, referring to expert advice, those factors were further summarized to be comprehensive, systematical, measurable and mutually exclusive in connotation by integrating elements with similar connotations, unifying variable names, modifying connotation statements. The final system of factors and their connotations (see in Table 1) has been formed into an academic paper published in the *China Pharmacy*(1).

Compiling influencing factors measurement questionnaire requires that all influencing factors are concrete and measurable. For the objective reality that can be directly measured, such as age, region, hospital grade, educational background, the questions and response options were prepared according to the research needs. For variables that cannot be directly measured, existing applicable scales were adopted. The measurement tools of influencing factors involved in this study are listed in the following table:

**Table 1 The 3-characteristic system of influencing factors and corresponding connotations**

| **Characteristics** | **Independent Variable** | **Connotations** | **Measurement Instruments** | **Number of Items** | **Remarks** |
| --- | --- | --- | --- | --- | --- |
| Individual Characteristic | 1-1 Gender | / | objective question | 1 |  |
|  | 1-2 Age | One of the objective representations of the participant's physical and mental state. | objective question | 1 |  |
|  | 1-3 Years in Practice | One of the objective representations of the accumulated work experience of participants. | objective question | 1 |  |
|  | 1-4 Location | An indicator of different levels of economic and social development and patients’ needs on clinical multidisciplinary collaboration. | objective question | 1 |  |
|  | 1-5 Area of Practice | An indicator of different levels of difficulty of rational drug use and needs on professional pharmaceutical care and collaboration. | objective question | 1 |  |
|  | 1-6 Type of Hospital | An indicator of different levels of difficulty of rational drug use and needs on professional pharmaceutical care and collaboration. | objective question | 1 |  |
|  | 1-7 Level of hospital |  |  |  |  |
|  | 1-7-1 Grade of hospital | An indicator of different needs and levels of the construction of clinical disciplines. | objective question | 1 |  |
|  | 1-7-2 Clinical Pharmacist Training Base | One of the objective representations of different development levels of clinical pharmacy. | objective question | 1 |  |
|  | 1-8 Personnel Status |  |  |  |  |
|  | 1-8-1 Professional Title | One of the objective representations of participants' comprehensive clinical competence and managerial experience. | objective question | 1 |  |
|  | 1-8-2 Type of Employment | An assessment and employment mechanism with Chinese characteristics, whether on the regular payroll or not represents the job stability to a certain extent. | objective question | 1 |  |
|  | 1-8-3 Type of Practice | One of the objective representations of the breadth and depth of clinical pharmacists participating in clinical work. | objective question | 2 |  |
|  | 1-9 Education and Training |  |  |  |  |
|  | 1-9-1 Terminal Degree | One of the objective representations of professional theoretical and practical ability of participants. | objective question | 1 |  |
|  | 1-9-2 Major | One of the objective representations of professional theoretical and practical ability of clinical pharmacists. | objective question | 1 |  |
|  | 1-9-3 Pattern of Training | One of the objective representations of professional theoretical and practical ability of clinical pharmacists. | objective question | 1 |  |
|  | 1-10 Interprofessional Background |  |  |  |  |
|  | 1-10-1 Interdisciplinary Education Experience | An indicator of whether clinical pharmacists and physicians have received versatile education in medicine, pharmacy and management science. | objective question | 1 |  |
|  | 1-10-2 Inter-career Working Experience | An indicator of whether clinical pharmacists and physicians have experience in different occupations. | objective question | 1 |  |
|  | 1-10-3 Interprofessional Collaboration Experience | An indicator of whether clinical pharmacists and physicians have good and rich experience in collaborative work. | objective question | 1 |  |
|  | 1-11 Personality |  |  |  |  |
|  | 1-11-1 Extraversion | An indicator of whether clinical pharmacists and physicians are outgoing and sociable. | 10-Item Big Five Inventory (BFI-10) by Beatrice Rammstedt et al. (2006) (2) | 10 | Richard Carciofo et al. (2016) (3) applied this scale in the Chinese population, and the Cronbach alpha coefficients of the five dimensions were 0.752, 0.037, 0.462, 0.628 and 0.438. |
|  | 1-11-2 Agreeableness | An indicator of whether clinical pharmacists and physicians are easygoing and reliable. |  |  |  |
|  | 1-11-3 Conscientiousness | An indicator of whether clinical pharmacists and physicians are responsible and persistent. |  |  |  |
|  | 1-11-4 Neuroticism | An indicator of whether clinical pharmacists and physicians are emotionally stable. |  |  |  |
|  | 1-11-5 Openness | An indicator of whether clinical pharmacists and physicians are open, inclusive and innovative. |  |  |  |
| Context Characteristic | 2-1 Institution Administration |  |  |  |  |
|  | 2-1-1 System Construction | An indicator of whether the hospital can provide perfect system guarantee for the collaboration between clinical pharmacists and physicians, such as work flow, performance appraisal, etc. | the long-form Minnesota Satisfaction Scale | 3 |  |
|  | 2-1-2 Manager Support | An indicator of whether hospital administrators attach importance to the collaboration between clinical pharmacists and physicians. |  | 3 |  |
|  | 2-1-3 Personnel Development | An indicator of whether the hospital can provide channels and platforms for further study, professional skills training, interprofessional training and other for clinical pharmacists or physicians. |  | 3 |  |
|  | 2-2 Job Satisfaction |  |  |  |  |
|  | 2-2-1 Workload | An indicator of whether clinical pharmacists or physicians suffer from heavy workload and lack of time. | the long-form Minnesota Satisfaction Scale | 2 |  |
|  | 2-2-2 Working Environment | An indicator of whether clinical pharmacists and physicians have a suitable and convenient office environment. |  | 2 |  |
|  | 2-2-3 Remuneration | An indicator of whether clinical pharmacists and physicians can obtain satisfactory salary and benefits. |  | 2 |  |
|  | 2-3 Working Atmosphere |  |  |  |  |
|  | 2-3-1 Team Acceptability | An indicator of whether the atmosphere within the team is harmonious and whether its members’ values, norms and beliefs are similar. | Team Psychological Security Climate Questionnaire by Wu Zhiping et al. (2011) (4) | 8 |  |
|  | 2-3-2 Social Acceptability | An indicator of whether the medical staff, patients and the masses have a full understanding on the profession of clinical pharmacists, physicians and interprofessional collaboration. | Professional Identification Scale (PIS) for nurses developed by Liu Ling et al. (2011) (5) | 5 |  |
|  | 2-4 Resources and Conditions |  |  |  |  |
|  | 2-4-1 Personnel Allocation | An indicator of whether the number of clinical pharmacists can meet clinical requirements. | objective question | 1 |  |
|  | 2-4-2 Facilities and Equipment | An indicator of whether the hardware and software equipment needed for collaboration between clinical pharmacists and physicians are well configured. | objective question | 2 |  |
|  | 2-5 Collaboration Incentives | An indicator of whether there is a clear payment or compensation mechanism for clinical pharmacist services and collaborative work. | objective question | 1 |  |
| Exchange Characteristic | 3-1 Respect | An indicator of whether clinical pharmacists and physicians can freely and directly propose or accept feedback in collaboration, and the independence of their work is guaranteed. | Job-Esteem Scale for Nurses in Hospital (JES-HN) by Choi et al. (2020) (6) | 4 | Shi Xiaopu et al. (2021) (7, 8) translated and adjusted across-culture into Chinese version |
|  | 3-2 Trust | An indicator of a clinical pharmacist's ability to gain the trust of a physician in his or her professional competence. | Organizational Trust Scale developed by McAllister (1995) (9) | 4 | Chua et al. (2008) (10) translated into Chinese version |
|  | 3-3 Role Recognition | An indicator of whether clinical pharmacists and physicians can fully recognize the professional responsibilities, limitations and boundaries of both parties to avoid role conflict and identity dilution. | Role Conflict and Ambiguity Scale (RHL) by Rizzo et al. (1970) (11) | 4 | Mei Hua et al. (2015)(12) translated into Chinese version |
|  | 3-4 Fairness | An indicator of whether clinical pharmacists and physicians believes that his effort is matched by his return | Organizational Justice Scale developed by Colquitt (2001) (13) | 3 |  |
|  | 3-5 Attitude to Collaboration |  |  |  |  |
|  | 3-5-1 Job Burnout | An indicator of whether clinical pharmacists and physicians can maintain stable and lasting career interest and avoid job burnout or lack of achievement | Maslach Burnout Inventory-general survey (MBI-GS) | 4 |  |
|  | 3-5-2 Usefulness Perception | An indicator of whether clinical pharmacists and physicians can recognize the value of collaboration in reducing clinical burden, improving treatment quality, and reducing costs | Self-developed questions | 1 |  |
|  | 3-5-3 Shared Expectation | An indicator of whether clinical pharmacists and physicians focus on the shared responsibility of collaboration, and uphold the belief that collaborative practice will improve patient care. | Self-developed questions | 2 |  |
|  | 3-6 Leadership |  |  |  |  |
|  | 3-6-1 Power Influence | An indicator of whether clinical pharmacists or physicians has a higher rank in the team, assuming leadership and participating in the work assessment of the other side | objective question | 1 |  |
|  | 3-6-2 Non-Power Influence | An indicator of whether clinical pharmacists or physicians are attractive in terms of good moral feeling or intellectual ability | objective question | 2 |  |
|  | 3-7 Familiarity |  |  |  |  |
|  | 3-7-1 Member Mobility | An indicator of whether clinical pharmacists and physicians can maintain consistent work schedules to reduce team member mobility and avoid fragmentation of patient care. | Lima (1998) (14) | 1 |  |
|  | 3-7-2 Out-of-Work Socializing | An indicator of whether clinical pharmacists and physicians can form a good personal level of social contact. | the Chinese supervisor–subordinate guanxi questionnaire by Law et al. (2011) (15) | 5 |  |
|  | 3-8 Communication |  |  |  |  |
|  | 3-8-1 Team Communication Ability | An indicator of clinical pharmacists' ability to communicate consistently and effectively with physicians about the treatment of patients. | Nurses' Clinic Communication Competency Scale compiled by Zeng Kai (2010) (16) | 4 |  |
|  | 3-8-2 General Language Ability | An indicator of whether clinical pharmacists can master clinical communication skills and understand physicians' communication preferences. |  | 9 |  |

**Reference**

1. Wang CY, Huang YK, Xi XY. Exploration of influential factors for collaboration between clinical pharmacists and physicians in China based on literature review. Chin Pharm. 2022;33(22):2791-2796.

2. Rammstedt B, John OP. Measuring personality in one minute or less: A 10-item short version of the Big Five Inventory in English and German. J Res Pers. 2007;41(1):203-212.

3. Carciofo R, Yang J, Song N, Du F, Zhang K. Psychometric evaluation of Chinese-language 44-Item and 10-Item Big Five Personality Inventories, including correlations with chronotype, mindfulness and mind wandering. PLoS ONE. 2016;11(2):e0149963.

4. Wu ZP, Chen FT. The scale development of team psychological safety climate in Chinese cultural context. Chin J Manage. 2011;8(1):73-80.

5. Liu L, Hao YF, Liu XH. Development of Professional Identity Scale for Nurses. Nurs J Chin PLA. 2011;28(3):18-20.

6. Choi HJ, Jung KI. Development of Job-Esteem Scale for Korean Nurses. J Korean Acad Nurs. 2020;50(3):444-458.

7. Shi XP, Shen XD. Chinese version of Job⁃Esteem Scale for Nurses in Hospital and its reliability and validity test Chin Nurs Res. 2021;35(19):3407-3411.

8. Shi XP, Li Y, Zhang CC. Correlation among job⁃esteem, job burnout and turnover intention in nurses employee turnover. Chin Nurs Res. 2021;35(15):2654-2660.

9. Mcallister DJ. Affect- and cognition-based trust as foundations for interpersonal cooperation in organizations. Acad Manage J. 1995;38:24-59.

10. Chua R, Ingram P, Morris MW. From the head and the heart: Locating cognition- and affect-based trust in managers' professional Networks. Acad Manage J. 2008;5(3):436-452.

11. Rizzo JR, House RJ, Lirtzman SI. Role conflict and ambiguity in complex organizations. Admin Sci Q. 1970;15(2):150-163.

12. Mei H, Zhang Y, Qin X, Sun Q. The reliability and validity of Chinese version of Nurse's Role Conflict and Ambiguity Scale. J Nurs Admin. 2015;15(1):3-5.

13. Colquitt JA. On the dimensionality of organizational justice: a construct validation of a measure. J Appl Psychol. 2001;86(3):386-400.

14. Lima JÁd. Improving the study of teacher collegiality: Methodological issues. 1998.

15. Law KS, Wong CS, Wang D, Wang L. Effect of supervisor–subordinate guanxi on supervisory decisions in China: an empirical investigation. Int J Hum Resour Manage. 2000;11(4):751-765.

16. Zeng K. The development and utilizatoin of Nurses' Clinic Communication Comptency Scale. China: Central South University; 2010.

**Supplement 2 Results of descriptive statistics**

**Table Results of descriptive statistics of all variables**

| **Variables** | **Clinical Pharmacist** | **Physician** |
| --- | --- | --- |
|  | **N (%) / Mean ± SD** | **N (%) / Mean ± SD** |
| 1-1 Gender |  |  |
| Male | 198 (41.77) | 264 (53.23) |
| Female | 276 (58.23) | 232 (46.77) |
| 1-2 Age | 38.85 ± 7.30 | 42.13 ± 7.71 |
| 1-3 Years in Practice | 11.01 ± 6.81 | 15.16 ± 7.64 |
| 1-4 Location |  |  |
| Eastern Region | 202 (42.62) | 215 (43.35) |
| Central Region | 121 (25.53) | 127 (25.60) |
| Western Region | 151 (31.86) | 154 (31.05) |
| 1-5 Area of Practice |  |  |
| General Department | 152 (32.07) | 43 (8.67) |
| Internal Medicine Department | 180 (37.97) | 135 (27.22) |
| Surgery Department | 56 (11.81) | 78 (15.73) |
| Gynecology Department | 36 (7.59) | 45 (9.07) |
| Pediatrics Department | 50 (10.55) | 49 (9.88) |
| Emergency and Critical Care Department ^1^ | 72 (15.19) | 67 (13.51) |
| Others ^2^ | 107 (22.57) | 116 (23.39) |
| 1-6 Type of hospital |  |  |
| General Hospital | 409 (86.29) | 430 (86.69) |
| Specialized Hospital | 65 (13.71) | 66 (13.31) |
| 1-7-1 Grade of hospital |  |  |
| Tertiary Hospital | 271 (57.17) | 277 (55.85) |
| Secondary Hospital | 203 (42.83) | 219 (44.15) |
| 1-7-2 Clinical Pharmacist Training Base |  |  |
| Yes | 219 (46.20) | —— |
| No | 255 (53.80) | —— |
| 1-8-1 Professional Title |  |  |
| Junior Title | 201 (42.41) | 74 (14.92) |
| Intermediate Title | 159 (33.54) | 227 (45.77) |
| Associate Senior Title | 76 (16.03) | 125 (25.20) |
| Senior Title | 38 (8.02) | 70 (14.11) |
| 1-8-2 Type of Employment |  |  |
| Regular Employee | 375 (79.11) | 421 (84.88) |
| Non-regular Employee | 99 (20.89) | 75 (15.12) |
| 1-8-3 Type of Practice ^*^ |  |  |
| Full-time Clinical Pharmacist | 432 (91.14) | 465 (93.75) |
| Part-time Clinical Pharmacist | 42 (8.86) | 31 (6.25) |
| Specialist Clinical Pharmacist | 215 (45.36) | 206 (41.53) |
| General Clinical Pharmacist | 259 (54.64) | 290 (58.47) |
| 1-9-1 Terminal Degree |  |  |
| Junior College Degree or below | 21 (4.43) | 7 (1.41) |
| Bachelor Degree | 226 (47.68) | 198 (39.92) |
| Master Degree | 198 (41.77) | 229 (46.17) |
| Doctor Degree or above | 29 (6.12) | 62 (12.50) |
| 1-9-2 Major |  |  |
| Clinical Pharmacy | 291 (61.39) | —— |
| Other Pharmacy-allied Majors | 160 (33.76) | —— |
| Non-pharmaceutical Related Majors | 23 (4.85) | —— |
| 1-9-3 Pattern of Training |  |  |
| Training after Graduation | 296 (62.45) | —— |
| Training after Job Transfer | 168 (35.44) | —— |
| Direct Assignment without Training | 10 (2.11) | —— |
| 1-10-1 Interdisciplinary Education Experience |  |  |
| Have Taken Basic Medical/Pharmaceutical Courses |  |  |
| Yes | 352 (74.26) | 409 (82.46) |
| No | 122 (25.74) | 87 (17.54) |
| Have Taken Management Science Courses |  |  |
| Yes | 403 (85.02) | 395 (79.64) |
| No | 71 (14.98) | 101 (20.36) |
| 1-10-2 Inter-career Working Experience |  |  |
| Yes | 86 (18.14) | 51 (10.28) |
| No | 388 (81.86) | 445 (89.72) |
| 1-10-3 Interprofessional Collaboration Experience | 5.97 ± 1.99 | 6.50 ± 0.35 |
| 1-11 Personality |  |  |
| 1-11-1 Extraversion | 3.42 ± 1.02 | 3.51 ± 0.99 |
| 1-11-2 Agreeableness | 3.76 ± 0.72 | 3.65 ± 0.76 |
| 1-11-3 Conscientiousness | 4.06 ± 0.66 | 4.10 ± 0.67 |
| 1-11-4 Neuroticism | 3.58 ± 0.80 | 3.66 ± 0.79 |
| 1-11-5 Openness | 3.45 ± 0.81 | 3.44 ± 0.81 |
| 2-1 Institution Administration |  |  |
| 2-1-1 System Construction | 21.04 ± 3.28 | 21.87 ± 2.74 |
| 2-1-2 Manager Support | 19.76 ± 3.50 | 20.21 ± 3.29 |
| 2-1-3 Personnel Development | 20.33 ± 3.08 | 21.24 ± 2.75 |
| 2-2 Job Satisfaction |  |  |
| 2-2-1 Workload | 7.69 ± 1.32 | 5.87 ± 2.74 |
| 2-2-2 Working Environment | 5.43 ± 1.81 | 7.65 ± 3.29 |
| 2-2-3 Remuneration | 7.03 ± 1.72 | 7.27 ± 2.75 |
| 2-3 Working Atmosphere |  |  |
| 2-3-1 Team Acceptability | 23.85 ± 4.31 | 23.58 ± 4.33 |
| 2-3-2 Social Acceptability | 4.05 ± 4.05 | 4.32 ± 0.50 |
| 2-4 Resources and Conditions |  |  |
| 2-4-1 Adequate Personnel Allocation |  |  |
| Yes | 211 (44.51) | 280 (56.45) |
| No | 263 (55.49) | 216 (43.55) |
| 2-4-2 Numbers of Needed Facilities and Equipment | 9.13 ± 3.41 | 4.17 ± 1.87 |
| 2-5 Incentives of Collaboration |  |  |
| Yes | 189 (39.87) | 178 (35.89) |
| No | 285 (60.13) | 318 (64.11) |
| 3-1 Respect | 4.03 ± 0.65 | 4.29 ± 0.52 |
| 3-2 Trust | 4.35 ± 0.57 | 4.40 ± 0.54 |
| 3-3 Role Recognition | 4.30 ± 0.49 | 4.36 ± 0.50 |
| 3-4 Fairness | 3.86 ± 0.64 | 11.82 ± 2.10 |
| 3-5 Attitude to Collaboration |  |  |
| 3-5-1 Job Burnout | 6.94 ± 3.56 | 6.85 ± 3.84 |
| 3-5-2 Perceived Usefulness | 5.26 ± 1.81 | 5.16 ± 1.82 |
| 3-5-3 Shared Expectation | 12.57 ± 1.48 | 12.56 ± 1.76 |
| 3-6 Leadership |  |  |
| 3-6-1 Power Influence |  |  |
| Same Office Rank | 223 (47.05) | 246 (49.60) |
| Higher Rank of Physician | 222 (46.84) | 199 (40.12) |
| Higher Rank of Clinical Pharmacist | 29 (6.12) | 51 (10.28) |
| 3-6-2 Non-Power Influence |  |  |
| Effect of Morality and Sentiment |  |  |
| Yes | 302 (63.71) | 309 (62.30) |
| No | 172 (36.29) | 187 (37.70) |
| Effect of Knowledge and Ability |  |  |
| Yes | 447 (94.30) | 478 (96.37) |
| No | 27 (5.70) | 18 (3.63) |
| 3-7 Familiarity |  |  |
| 3-7-1 Member Mobility | 3.66 ± 0.77 | 3.59 ± 0.85 |
| 3-7-2 Out-of-Work Socializing | 3.22 ± 0.91 | 3.18 ± 0.94 |
| 3-8 Communication |  |  |
| 3-8-1 Team Communication Ability | 4.26 ± 0.53 | 4.35 ± 0.58 |
| 3-8-2 General Language Ability | 4.26 ± 0.52 | 4.29 ± 0.57 |
|  |  |  |
| Pharmacist-physician Collaboration Index Scale |  |  |
| Total Score | 86.84 ± 10.81 | 86.19 ± 11.81 |
| Trustworthiness | 38.11 ± 4.79 | 37.74 ± 5.05 |
| Role Specification | 29.86 ± 4.82 | 30.02 ± 4.83 |
| Relationship Initiation | 18.87 ± 2.47 | 18.42 ± 2.72 |

Note:

^1^“Emergency and Critical Care Departments” include Emergency Medicine Department, Respiratory and Critical Care Medicine Department, Infection Department and Oncology Department;

^2^“Others” include Ophthalmology Department, Otolaryngology Department, Stomatology Department, Dermatology Department, Anesthesiology Department, Rehabilitation Medicine Department, Department of Traditional Chinese Medicine, Department of Geriatrics, Psychiatry Department, Department of Pain Treatment and Intervention Department;

“-” means this factor is only measured for clinical pharmacists. See Appendix 4 for the complete questionnaire;

“*” indicates that this variable has different connotation in clinical pharmacist questionnaire and physician questionnaire. See Appendix 4 for the complete questionnaire.

**Supplement 3 Results of normality, reliability, collinearity, correlation and robustness analysis**

3.1 Table 1 Normality test of the sample data of clinical pharmacists and physicians

| **Variables** | **Clinical Pharmacists** | | | **Physicians** | | |
| --- | --- | --- | --- | --- | --- | --- |
|  | **Skewness** | **Kurtosis** | **P-value of SW-Test*** | **Skewness** | **Kurtosis** | **P-value of SW-Test** |
| PPCI Sum | -2.43 | 12.25 | 0.00 | -2.08 | 7.77 | 0.00 |
| 1-2 Age | 0.50 | 3.44 | 0.00 | 0.20 | 3.26 | 0.16 |
| 1-3 Years in Practice | 0.95 | 3.57 | 0.00 | 0.47 | 3.09 | 0.00 |
| 1-11-1 Extraversion | -0.35 | 2.10 | 0.00 | -0.39 | 2.18 | 0.00 |
| 1-11-2 Agreeableness | -0.16 | 2.49 | 0.08 | -0.14 | 2.37 | 0.02 |
| 1-11-3 Conscientiousness | -0.48 | 2.90 | 0.00 | -0.56 | 3.04 | 0.00 |
| 1-11-4 Neuroticism | -0.30 | 2.63 | 0.01 | -0.28 | 2.44 | 0.02 |
| 1-11-5 Openness | -0.17 | 2.46 | 0.32 | -0.22 | 2.96 | 0.07 |
| 2-1-1 System Construction | -0.90 | 4.46 | 0.00 | -0.63 | 3.71 | 0.00 |
| 2-1-2 Manager Support | -0.39 | 2.84 | 0.00 | -0.26 | 2.60 | 0.00 |
| 2-1-3 Personnel Development | -0.42 | 3.42 | 0.00 | -0.24 | 2.66 | 0.00 |
| 2-2-1 Workload | 0.07 | 2.53 | 0.26 | -0.09 | 2.32 | 0.02 |
| 2-2-2 Working Environment | -0.80 | 4.70 | 0.00 | -0.88 | 4.60 | 0.00 |
| 2-2-3 Remuneration | -0.87 | 2.96 | 0.00 | -1.02 | 3.47 | 0.00 |
| 2-3-1 Team Acceptability | 1.09 | 6.14 | 0.00 | 0.85 | 4.57 | 0.00 |
| 2-3-2 Social Acceptability | -0.39 | 3.59 | 0.00 | -0.31 | 3.62 | 0.00 |
| 3-1 Respect | -0.44 | 3.38 | 0.00 | -0.16 | 3.22 | 0.00 |
| 3-2 Trust | -0.73 | 5.02 | 0.00 | -0.60 | 3.80 | 0.00 |
| 3-3 Role Recognition | -0.02 | 2.58 | 0.00 | -0.15 | 2.35 | 0.00 |
| 3-4 Fairness | -0.78 | 4.51 | 0.00 | -0.93 | 4.58 | 0.00 |
| 3-5-1 Job Burnout | 2.18 | 6.67 | 0.00 | 2.51 | 8.38 | 0.00 |
| 3-5-2 Perceived Usefulness | -0.79 | 2.50 | 0.00 | -0.65 | 2.17 | 0.00 |
| 3-5-3 Shared Expectation | -1.53 | 4.40 | 0.00 | -2.39 | 9.46 | 0.00 |
| 3-7-1 Member Mobility | -0.28 | 3.50 | 0.00 | -0.39 | 3.58 | 0.00 |
| 3-7-2 Out-of-Work Socializing | -0.13 | 2.68 | 0.67 | -0.16 | 2.74 | 0.25 |
| 3-8-1 Team Communication Ability | -0.08 | 2.57 | 0.00 | -0.50 | 2.86 | 0.00 |
| 3-8-2 General Language Ability | -0.18 | 2.50 | 0.00 | -0.43 | 2.73 | 0.00 |

Note:

* SW-Test means Shapiro-Wilk Test. Generally speaking, P-value＞0.05 indicates that the variable conforms to a normal distribution.

3.2 Table 2 Reliability, collinearity, correlation analysis of the sample data of clinical pharmacists

| **Variables** | **Pre-α** | **α** | **VIF** | **(1)** | **(2)** | **(3)** | **(4)** | **(5)** | **(6)** | **(7)** | **(8)** | **(9)** | **(10)** | **(11)** | **(12)** | **(13)** | **(14)** | **(15)** | **(16)** | **(17)** | **(18)** | **(19)** | **(20)** | **(21)** | **(22)** | **(23)** | **(24)** | **(25)** |
| --- | --- | --- | --- | --- | --- | --- | --- | --- | --- | --- | --- | --- | --- | --- | --- | --- | --- | --- | --- | --- | --- | --- | --- | --- | --- | --- | --- | --- |
| (1) Y | 0.95 | 0.96 | - | 1.000 |  |  |  |  |  |  |  |  |  |  |  |  |  |  |  |  |  |  |  |  |  |  |  |  |
| (2) X1-11-1 | 0.87 | 0.82 | 1.39 | 0.188  *** | 1.000 |  |  |  |  |  |  |  |  |  |  |  |  |  |  |  |  |  |  |  |  |  |  |  |
| (3) X1-11-2 | 0.21 | 0.03 | 1.14 | 0.135  *** | 0.062 | 1.000 |  |  |  |  |  |  |  |  |  |  |  |  |  |  |  |  |  |  |  |  |  |  |
| (4) X1-11-3 | 0.42 | 0.45 | 1.58 | 0.321  *** | 0.342  *** | 0.156  *** | 1.000 |  |  |  |  |  |  |  |  |  |  |  |  |  |  |  |  |  |  |  |  |  |
| (5) X1-11-4 | 0.66 | 0.46 | 1.64 | 0.206  *** | 0.464  *** | 0.176  *** | 0.452  *** | 1.000 |  |  |  |  |  |  |  |  |  |  |  |  |  |  |  |  |  |  |  |  |
| (6) X1-11-5 | 0.37 | 0.34 | 1.18 | 0.207  *** | 0.205  *** | 0.142  *** | 0.264  *** | 0.213  *** | 1.000 |  |  |  |  |  |  |  |  |  |  |  |  |  |  |  |  |  |  |  |
| (7) X2-1-1 | 0.92 | 0.89 | 2.41 | 0.393  *** | 0.242  *** | 0.052 | 0.225  *** | 0.200  *** | 0.098  ** | 1.000 |  |  |  |  |  |  |  |  |  |  |  |  |  |  |  |  |  |  |
| (8) X2-1-2 | 0.87 | 0.88 | 3.19 | 0.418  *** | 0.205  *** | -0.007 | 0.196  *** | 0.203  *** | 0.152  *** | 0.673  *** | 1.000 |  |  |  |  |  |  |  |  |  |  |  |  |  |  |  |  |  |
| (9) X2-1-3 | 0.84 | 0.85 | 3.11 | 0.398  *** | 0.223  *** | 0.037  *** | 0.230 | 0.165  *** | 0.170  *** | 0.698  *** | 0.761  *** | 1.000 |  |  |  |  |  |  |  |  |  |  |  |  |  |  |  |  |
| (10) X2-2-1 | 0.97 | 0.82 | 1.44 | -0.156  *** | -0.192  *** | -0.065 | -0.309  *** | -0.309  *** | -0.085  * | -0.267  *** | -0.257  *** | -0.206  *** | 1.000 |  |  |  |  |  |  |  |  |  |  |  |  |  |  |  |
| (11) X2-2-2 | 0.81 | 0.78 | 1.47 | 0.268  *** | 0.227 | 0.061 | 0.234  *** | 0.220  *** | 0.061 | 0.349  *** | 0.339  *** | 0.351  *** | -0.317  *** | 1.000 |  |  |  |  |  |  |  |  |  |  |  |  |  |  |
| (12) X2-2-3 | 0.71 | 0.87 | 2.08 | 0.233  *** | 0.157  *** | 0.041 | 0.278  *** | 0.175  *** | 0.090  ** | 0.335  *** | 0.339  *** | 0.290  *** | -0.399  *** | 0.406  *** | 1.000 |  |  |  |  |  |  |  |  |  |  |  |  |  |
| (13) X2-3-1 | 0.80 | 0.70 | 1.23 | 0.128  *** | 0.068 | 0.047 | 0.232  *** | 0.173  *** | 0.136  *** | 0.049 | -0.034 | -0.012 | -0.202  *** | -0.011 | 0.114  ** | 1.000 |  |  |  |  |  |  |  |  |  |  |  |  |
| (14) X2-3-2 | 0.82 | 0.85 | 2.04 | 0.391  *** | 0.217  *** | 0.051 | 0.279  *** | 0.173  *** | 0.159  *** | 0.467  *** | 0.554  *** | 0.529  *** | -0.182  *** | 0.357  *** | 0.290  *** | -0.022 | 1.000 |  |  |  |  |  |  |  |  |  |  |  |
| (15) X3-1 | 0.96 | 0.89 | 3.31 | 0.453  *** | 0.228  *** | 0.066 | 0.262  *** | 0.230  *** | 0.187  *** | 0.586  *** | 0.673  *** | 0.637  *** | -0.267  *** | 0.396  *** | 0.394  *** | -0.018 | 0.665  *** | 1.000 |  |  |  |  |  |  |  |  |  |  |
| (16) X3-2 | 0.97 | 0.93 | 2.31 | 0.394  *** | 0.230  *** | 0.120  *** | 0.252  *** | 0.232  *** | 0.142  *** | 0.516  *** | 0.453  *** | 0.517  *** | -0.233  *** | 0.257  *** | 0.263  *** | 0.102  ** | 0.503  *** | 0.641  *** | 1.000 |  |  |  |  |  |  |  |  |  |
| (17) X3-3 | 0.81 | 0.86 | 2.14 | 0.388  *** | 0.240  *** | 0.161  *** | 0.321  *** | 0.330  *** | 0.204  *** | 0.463  *** | 0.444  *** | 0.492  *** | -0.237  *** | 0.312  *** | 0.257  *** | 0.035 | 0.506  *** | 0.572  *** | 0.582  *** | 1.000 |  |  |  |  |  |  |  |  |
| (18) X3-5-1 | 0.83 | 0.92 | 1.59 | -0.270  *** | -0.209  *** | -0.180  *** | -0.367  *** | -0.291  *** | -0.181  *** | -0.318  *** | -0.271  *** | -0.289  *** | 0.263  *** | -0.258  *** | -0.349  *** | -0.233  *** | -0.296  *** | -0.378  *** | -0.288  *** | -0.294  *** | 1.000 |  |  |  |  |  |  |  |
| (19) X3-5-2 | - | - | 1.26 | 0.244  *** | 0.095  ** | 0.073 | 0.199  *** | 0.171  *** | 0.125  *** | 0.200  *** | 0.257  *** | 0.232  *** | -0.204  *** | 0.192  *** | 0.210  *** | 0.151  *** | 0.231  *** | 0.234  *** | 0.304  *** | 0.253  *** | -0.196  *** | 1.000 |  |  |  |  |  |  |
| (20) X3-5-3 | 0.80 | 0.74 | 1.80 | 0.380  *** | 0.216  *** | 0.150  *** | 0.362  *** | 0.251  *** | 0.174  *** | 0.345  *** | 0.332  *** | 0.362  *** | -0.182  *** | 0.278  *** | 0.248  *** | 0.094  ** | 0.399  *** | 0.501  *** | 0.460  *** | 0.413  *** | -0.491  *** | 0.333  *** | 1.000 |  |  |  |  |  |
| (21) X3-4 | 0.74 | 0.82 | 2.35 | 0.281  *** | 0.170  *** | 0.048 | 0.249  *** | 0.204  *** | 0.087  * | 0.462  *** | 0.457  *** | 0.436  *** | -0.430  *** | 0.427  *** | 0.647  *** | 0.051 | 0.364  *** | 0.502  *** | 0.434  *** | 0.418  *** | -0.322  *** | 0.247  *** | 0.299  *** | 1.000 |  |  |  |  |
| (22) X3-7-1 | - | - | 1.27 | 0.191  *** | 0.085  * | 0.078  * | 0.161  *** | 0.139  *** | 0.165  *** | 0.242  *** | 0.262  *** | 0.233  *** | -0.197  *** | 0.222  *** | 0.188  *** | 0.072 | 0.204  *** | 0.297  *** | 0.281  *** | 0.267  *** | -0.195  *** | 0.230  *** | 0.268  *** | 0.300  *** | 1.000 |  |  |  |
| (23) X3-7-2 | 0.90 | 0.90 | 1.40 | 0.202  *** | 0.162  *** | -0.021 | 0.135  *** | 0.133  *** | 0.153  *** | 0.252  *** | 0.298  *** | 0.296  *** | -0.218  *** | 0.307  *** | 0.331  *** | -0.098  ** | 0.248  *** | 0.317  *** | 0.205  *** | 0.275  *** | -0.138  *** | 0.152  *** | 0.156  *** | 0.352  *** | 0.310  *** | 1.000 |  |  |
| (24) X3-8-1 | 0.83 | 0.88 | 2.81 | 0.423  *** | 0.264  *** | 0.145  *** | 0.299  *** | 0.313  *** | 0.161  *** | 0.462  *** | 0.459  *** | 0.507  *** | -0.194  *** | 0.291  *** | 0.174  *** | 0.073 | 0.480  *** | 0.552  *** | 0.587  *** | 0.605  *** | -0.282  *** | 0.223  *** | 0.433  *** | 0.397  *** | 0.299  *** | 0.268  *** | 1.000 |  |
| (25) X3-8-2 | 0.95 | 0.92 | 2.50 | 0.414  *** | 0.224  *** | 0.212  *** | 0.240  *** | 0.233  *** | 0.155  *** | 0.437  *** | 0.405  *** | 0.478  *** | -0.187  *** | 0.305  *** | 0.148  *** | 0.029 | 0.404  *** | 0.475  *** | 0.511  *** | 0.575  *** | -0.224  *** | 0.227  *** | 0.366  *** | 0.371  *** | 0.244  *** | 0.320  *** | 0.727  *** | 1.000 |

3.3 Table 3 Reliability, collinearity, correlation analysis of the sample data of physician

| **Variables** | **Pre-α** | **α** | **VIF** | **(1)** | **(2)** | **(3)** | **(4)** | **(5)** | **(6)** | **(7)** | **(8)** | **(9)** | **(10)** | **(11)** | **(12)** | **(13)** | **(14)** | **(15)** | **(16)** | **(17)** | **(18)** | **(19)** | **(20)** | **(21)** | **(22)** | **(23)** | **(24)** | **(25)** |
| --- | --- | --- | --- | --- | --- | --- | --- | --- | --- | --- | --- | --- | --- | --- | --- | --- | --- | --- | --- | --- | --- | --- | --- | --- | --- | --- | --- | --- |
| (1) Y | 0.98 | 0.97 | - | 1.000 |  |  |  |  |  |  |  |  |  |  |  |  |  |  |  |  |  |  |  |  |  |  |  |  |
| (2) X1-11-1 | 0.86 | 0.81 | 1.48 | 0.139  *** | 1.000 |  |  |  |  |  |  |  |  |  |  |  |  |  |  |  |  |  |  |  |  |  |  |  |
| (3) X1-11-2 | 0.06 | 0.04 | 1.14 | 0.120  *** | -0.009 | 1.000 |  |  |  |  |  |  |  |  |  |  |  |  |  |  |  |  |  |  |  |  |  |  |
| (4) X1-11-3 | 0.66 | 0.38 | 1.53 | 0.156  *** | 0.324  *** | 0.140  *** | 1.000 |  |  |  |  |  |  |  |  |  |  |  |  |  |  |  |  |  |  |  |  |  |
| (5) X1-11-4 | 0.84 | 0.45 | 1.63 | 0.214  *** | 0.488  *** | 0.150  *** | 0.423  *** | 1.000 |  |  |  |  |  |  |  |  |  |  |  |  |  |  |  |  |  |  |  |  |
| (6) X1-11-5 | 0.65 | 0.24 | 1.19 | 0.115  ** | 0.304  *** | 0.075  * | 0.240  *** | 0.186  *** | 1.000 |  |  |  |  |  |  |  |  |  |  |  |  |  |  |  |  |  |  |  |
| (7) X2-1-1 | 0.94 | 0.90 | 3.00 | 0.513  *** | 0.140  *** | 0.127  *** | 0.292  *** | 0.275  *** | 0.116  *** | 1.000 |  |  |  |  |  |  |  |  |  |  |  |  |  |  |  |  |  |  |
| (8) X2-1-2 | 0.89 | 0.88 | 2.80 | 0.488  *** | 0.164  *** | 0.053 | 0.178  *** | 0.234  *** | 0.098** | 0.681*** | 1.000 |  |  |  |  |  |  |  |  |  |  |  |  |  |  |  |  |  |
| (9) X2-1-3 | 0.88 | 0.86 | 2.65 | 0.429  *** | 0.169  *** | 0.034 | 0.249  *** | 0.244  *** | 0.118  *** | 0.721  *** | 0.667  *** | 1.000 |  |  |  |  |  |  |  |  |  |  |  |  |  |  |  |  |
| (10) X2-2-1 | 0.79 | 0.84 | 1.60 | -0.131  *** | -0.187  *** | -0.007 | -0.140  *** | -0.232  *** | -0.096  ** | -0.198  *** | -0.294  *** | -0.207  *** | 1.000 |  |  |  |  |  |  |  |  |  |  |  |  |  |  |  |
| (11) X2-2-2 | 0.82 | 0.87 | 1.66 | 0.259  *** | 0.208  *** | 0.084  * | 0.260  *** | 0.251  *** | 0.168  *** | 0.392  *** | 0.438  *** | 0.389  *** | -0.410  *** | 1.000 |  |  |  |  |  |  |  |  |  |  |  |  |  |  |
| (12) X2-2-3 | 0.60 | 0.89 | 2.16 | 0.220  *** | 0.132  *** | -0.019 | 0.204  *** | 0.193  *** | 0.150  *** | 0.327  *** | 0.377  *** | 0.342  *** | -0.454  *** | 0.485  *** | 1.000 |  |  |  |  |  |  |  |  |  |  |  |  |  |
| (13) X2-3-1 | 0.86 | 0.72 | 1.33 | 0.058 | 0.161  *** | 0.094  ** | 0.255  *** | 0.206  *** | 0.124  *** | 0.088  * | 0.029 | 0.072 | -0.306  *** | 0.184  *** | 0.097  ** | 1.000 |  |  |  |  |  |  |  |  |  |  |  |  |
| (14) X2-3-2 | 0.93 | 0.87 | 2.66 | 0.388  *** | 0.153  *** | 0.100  ** | 0.300  *** | 0.267  *** | 0.130  *** | 0.575  *** | 0.561  *** | 0.606  *** | -0.208  *** | 0.397  *** | 0.361  *** | 0.048 | 1.000 |  |  |  |  |  |  |  |  |  |  |  |
| (15) X3-1 | 0.83 | 0.90 | 3.48 | 0.401  *** | 0.149  *** | 0.073 | 0.312  *** | 0.290  *** | 0.084  * | 0.661  *** | 0.657  *** | 0.635  *** | -0.226  *** | 0.387  *** | 0.352  *** | 0.058 | 0.727  *** | 1.000 |  |  |  |  |  |  |  |  |  |  |
| (16) X3-2 | 0.93 | 0.93 | 2.94 | 0.523  *** | 0.087* | 0.179  *** | 0.239  *** | 0.252  *** | 0.102  ** | 0.647  *** | 0.578  *** | 0.563  *** | -0.152  *** | 0.333  *** | 0.258  *** | 0.090  ** | 0.613  *** | 0.664  *** | 1.000 |  |  |  |  |  |  |  |  |  |
| (17) X3-3 | 0.90 | 0.87 | 2.19 | 0.391  *** | 0.161  *** | 0.104  ** | 0.352  *** | 0.314  *** | 0.098  ** | 0.591  *** | 0.506  *** | 0.533  *** | -0.182  *** | 0.339  *** | 0.319  *** | 0.102  ** | 0.612  *** | 0.643  *** | 0.608  *** | 1.000 |  |  |  |  |  |  |  |  |
| (18) X3-5-1 | 0.89 | 0.92 | 1.46 | -0.133  *** | -0.171  *** | -0.114  ** | -0.307  *** | -0.265  *** | -0.151  *** | -0.258  *** | -0.138  *** | -0.207  *** | 0.317  *** | -0.295  *** | -0.267  *** | -0.326  *** | -0.291  *** | -0.290  *** | -0.204  *** | -0.251  *** | 1.000 |  |  |  |  |  |  |  |
| (19) X3-5-2 | - | - | 1.31 | 0.373  *** | 0.138  *** | 0.072  *** | 0.192  *** | 0.164  *** | 0.051 | 0.244  *** | 0.281  *** | 0.185  *** | -0.133  *** | 0.202  *** | 0.198  *** | 0.119  *** | 0.242  *** | 0.254  *** | 0.345  *** | 0.301  *** | -0.129  *** | 1.000 |  |  |  |  |  |  |
| (20) X3-5-3 | 0.96 | 0.86 | 1.57 | 0.390  *** | 0.066 | 0.133  *** | 0.188  *** | 0.150  *** | 0.046 | 0.358  *** | 0.310  *** | 0.323  *** | -0.074  * | 0.139  *** | 0.156  *** | 0.038 | 0.404  *** | 0.356  *** | 0.481  *** | 0.323  *** | -0.238  *** | 0.344  *** | 1.000 |  |  |  |  |  |
| (21) X3-4 | 0.87 | 0.88 | 2.57 | 0.285  *** | 0.143  *** | 0.070 | 0.167  *** | 0.210  *** | 0.149  *** | 0.453  *** | 0.504  *** | 0.429  *** | -0.378  *** | 0.463  *** | 0.658  *** | 0.028 | 0.518  *** | 0.551  *** | 0.460  *** | 0.433  *** | -0.279  *** | 0.270  *** | 0.338  *** | 1.000 |  |  |  |  |
| (22) X3-7-1 | - | - | 1.45 | 0.247  *** | 0.069 | 0.061 | 0.173  *** | 0.086  * | 0.030 | 0.275  *** | 0.306  *** | 0.235  *** | -0.066 | 0.181  *** | 0.134  *** | 0.084  * | 0.245  *** | 0.305  *** | 0.377  *** | 0.242  *** | -0.167  *** | 0.281  *** | 0.297  *** | 0.296  *** | 1.000 |  |  |  |
| (23) X3-7-2 | 0.94 | 0.91 | 1.42 | 0.203  *** | 0.118  *** | -0.072 | 0.085  * | 0.116  *** | 0.125  *** | 0.213  *** | 0.294  *** | 0.252  *** | -0.217  *** | 0.256  *** | 0.338  *** | -0.058 | 0.254  *** | 0.254  *** | 0.246  *** | 0.201  *** | -0.117  *** | 0.187  *** | 0.105  ** | 0.358  *** | 0.369  *** | 1.000 |  |  |
| (24) X3-8-1 | 0.94 | 0.92 | 3.36 | 0.483  *** | 0.131  *** | 0.148  *** | 0.295  *** | 0.309  *** | 0.050 | 0.562  *** | 0.547  *** | 0.482  *** | -0.094  ** | 0.264  *** | 0.234  *** | 0.113  ** | 0.511  *** | 0.608  *** | 0.684  *** | 0.547  *** | -0.247  *** | 0.354  *** | 0.456  *** | 0.436  *** | 0.419  *** | 0.245  *** | 1.000 |  |
| (25) X3-8-2 | 0.97 | 0.94 | 3.35 | 0.522  *** | 0.130  *** | 0.186  *** | 0.293  *** | 0.300  *** | 0.096  ** | 0.556  *** | 0.579  *** | 0.519  *** | -0.119  *** | 0.262  *** | 0.246  *** | 0.038 | 0.558  *** | 0.647  *** | 0.636  *** | 0.550  *** | -0.178  *** | 0.355  *** | 0.466  *** | 0.433*** | 0.383  *** | 0.304  *** | 0.781  *** | 1.000 |

3.4 Table 4 The results of multiple stepwise regression

| **Clinical pharmacists** | | | | **Physicians** | | | |
| --- | --- | --- | --- | --- | --- | --- | --- |
| **Variables** | **Coef. (SE)** | **95% CI** | **T-test (p-value)** | **Variables** | **Coef. (SE)** | **95% CI** | **T-test (p-value)** |
| 1-7-1 Grade of Hospital (Base: Secondary Hospital) | | | | 1-4 Location (Base: Eastern Region) | | | |
| Tertiary Hospital | -1.847 (0.839) | -3.496 (-0.198) | **-2.20 (0.028**)** | Central Region | 2.233 (0.941) | 0.383 (4.083) | **2.37 (0.018**)** |
| 1-9-1 Terminal Degree (Base: Junior College Degree or below) | | | | 1-5 Area of Practice | | | |
| Bachelor Degree | -1.458 (0.831) | -3.091 (0.175) | -1.75 (0.080*) | Gynecology Department | -3.163 (1.406) | -5.925 (-0.400) | **-2.25 (0.025**)** |
| 1-9-3 Pattern of Training (Base: Training after Graduation) | | | | Pediatrics Department | -2.705 (1.355) | -5.368 (-0.043) | **-2.00 (0.046**)** |
| Direct Assignment without Training | -9.526 (2.837) | -15.101 (-3.951) | -**3.36 (0.001***)** | 1-6 Type of Hospital (Base: Specialized Hospital) | | | |
| 1-11 Personality | | | | General Hospital | 2.310 (1.200) | -0.048 (4.667) | 1.92 (0.055*) |
| 1-11-3 Conscientiousness | 2.394 (0.688) | 1.043 (3.745) | **3.48 (0.001***)** | 1-7-1 Grade of Hospital (Base: Secondary Hospital) | | | |
|  |  |  |  | Tertiary Hospital | -2.157 (0.824) | -3.775 (-0.538) | **-2.62 (0.009***)** |
|  |  |  |  | 1-11 Personality | | | |
|  |  |  |  | 1-11-1 Extraversion | 0.729 (0.434) | -0.122 (1.581) | 1.68 (0.093*) |
|  |  |  |  | 1-11-3 Conscientiousness | -1.190 (0.681) | -2.528 (0.147) | -1.75 (0.081*) |
| 2-1 Institution Administration |  |  |  | 2-1 Institution Administration | | | |
| 2-1-2 Manager Support | 2.827 (0.803) | 1.248 (4.405) | **3.52 (0.000***)** | 2-1-1 System Construction | 5.542 (1.157) | 3.269 (7.816) | **4.79 (0.000***)** |
| 2-3 Working Atmosphere |  |  |  | 2-1-2 Manager Support | 2.236 (0.947) | 0.375 (4.097) | **2.36 (0.019**)** |
| 2-3-1 Team Acceptability | 0.196 (0.098) | 0.004 (0.388) | **2.00 (0.046**)** | 2-2 Job Satisfaction | | | |
| 2-5 Collaboration Incentives (Base: No) | -1.578 (0.888) | -3.323 (0.166) | -1.78 (0.076*) | 2-2-3 Remuneration | 1.291 (0.658) | -0.001 (2.583) | **1.96 (0.050**)** |
| 3-1 Respect | 2.375 (0.953) | 0.502 (4.248) | **2.49 (0.013**)** | 3-1 Respect | -2.529 (1.285) | -5.055 (-0.003) | **-1.97 (0.050**)** |
| 3-5 Attitude to Collaboration |  |  |  | 3-2 Trust | 3.899 (1.165) | 1.611 (6.188) | **3.35 (0.001***)** |
| 3-5-3 Shared Expectation | 0.985 (0.334) | 0.329 (1.642) | **2.95 (0.003***)** | 3-4 Fairness | -2.496 (0.883) | -4.231 (-0.762) | **-2.83 (0.005***)** |
| 3-6 Leadership |  |  |  | 3-5 Attitude to Collaboration |  |  |  |
| 3-6-2 Non-Power Influence |  |  |  | 3-5-2 Usefulness Perception | 0.958 (0.245) | 0.476 (1.440) | **3.91 (0.000***)** |
| Effect of Morality and Sentiment (Base: No) | 1.572 (0.851) | -0.100 (3.245) | 1.85 (0.065*) | 3-5-3 Shared Expectation | 0.746 (0.275) | 0.205 (1.286) | **2.71 (0.007***)** |
| 3-8 Communication |  |  |  | 3-6 Leadership | | | |
| 3-8-2 General Language Ability | 4.140 (0.907) | 2.357 (5.924) | **4.56 (0.000***)** | 3-6-1 Power Influence (Base: Same Office Rank) | | | |
|  |  |  |  | Higher Rank of Physician | -2.156 (0.845) | -3.818 (-0.495) | **-2.55 (0.011**)** |
|  |  |  |  | 3-8 Communication | | | |
|  |  |  |  | 3-8-2 General Language Ability | 4.299 (1.056) | 2.223 (6.374) | **4.07 (0.000***)** |
| Cons. | 23.281 (4.712) | 14.022 (32.540) | 4.94 (0.000) | Cons. | 21.369(4.346) | 12.830 (29.908) | 4.92 (0.000) |
| R^2^ | 0.355 |  |  | R^2^ | 0.460 |  |  |
| Adjusted R^2^ | 0.339 |  |  | Adjusted R^2^ | 0.441 |  |  |

Note: *,**,*** indicate respectively P＜0.1，P＜0.05，P＜0.01. Variables with P>0.05 are bolded to facilitate reading.

3.5 Table 5 The results of robustness analysis

| **Variables** | **Clinical Pharmacist** | | | | **Physician** | | | |
| --- | --- | --- | --- | --- | --- | --- | --- | --- |
|  | **（1）** | **（2）** | **（3）** | **（4）** | **（1）** | **（2）** | **（3）** | **（4）** |
|  | **Standardized Coef.** | **Standardized Coef.** | **Standardized Coef.** | **Standardized Coef.** | **Standardized Coef.** | **Standardized Coef.** | **Standardized Coef.** | **Standardized Coef.** |
| 1-1 Gender (Base: Female) |  |  |  |  |  |  |  |  |
| Male | -0.007 | -0.015 | 0.060 | 0.008 | -0.004 | 0.004 | -0.005 | 0.004 |
| 1-2 Age | 0.032 | -0.019 | -0.018 | -0.043 | 0.018 | 0.065 | -0.027 | 0.005 |
| 1-3 Years in Practice | -0.068 | -0.024 | -0.132* | -0.032 | -0.006 | -0.095 | -0.004 | -0.016 |
| 1-4 Location (Base: Eastern Region) |  |  |  |  |  |  |  |  |
| Central Region | -0.059 | -0.045 | -0.030 | -0.034 | 0.088* | 0.073* | 0.074* | 0.070* |
| Western Region | -0.003 | 0.010 | -0.002 | 0.018 | 0.017 | -0.015 | 0.031 | 0.005 |
| 1-5 Area of Practice |  |  |  |  |  |  |  |  |
| General Department | -0.023 | -0.069 | -0.036 | -0.022 | 0.006 | -0.072 | -0.049 | -0.024 |
| Internal Medicine Department | -0.035 | -0.042 | -0.046 | -0.029 | -0.010 | -0.035 | -0.003 | -0.009 |
| Surgery Department | 0.010 | 0.012 | 0.025 | 0.018 | -0.008 | -0.011 | 0.004 | -0.002 |
| Gynecology Department | 0.069 | 0.034 | **0.101**** | 0.071* | -0.071 | -0.079 | -0.038 | -0.074 |
| Pediatrics Department | -0.030 | -0.026 | **-0.089**** | -0.067* | -0.042 | -0.093* | -0.064 | -0.055 |
| Emergency and Critical Care Department | 0.012 | -0.009 | -0.017 | -0.022 | -0.004 | -0.032 | -0.038 | -0.022 |
| Others | 0.040 | 0.001 | 0.057 | 0.039 | 0.065 | -0.030 | 0.008 | 0.017 |
| 1-6 Type of hospital (Base: Specialized Hospital) |  |  |  |  |  |  |  |  |
| General Hospital | 0.021 | 0.001 | 0.015 | 0.003 | 0.040 | 0.062 | 0.072* | 0.047 |
| 1-7-1 Grade of hospital (Base: Secondary Hospital) |  |  |  |  |  |  |  |  |
| Tertiary Hospital | -0.003 | **-0114**** | **-0.100**** | -0.042 | -0.080* | **-0.099**** | -0.079* | **-0.086**** |
| 1-7-2 Clinical Pharmacist Training Base (Base: No) | -0.004 | -0.017 | -0.016 | -0.020 |  |  |  |  |
| 1-8-1 Professional Title (Base: Junior Title) |  |  |  |  |  |  |  |  |
| Intermediate Title | -0.007 | 0.013 | -0.075 | 0.018 | 0.043 | 0.088 | 0.032 | 0.067 |
| Associate Senior Title | -0.001 | -0.008 | 0.036 | 0.024 | 0.090 | 0.097 | 0.048 | 0.084 |
| Senior Title | 0.029 | -0.033 | 0.011 | -0.010 | 0.043 | 0.064 | 0.047 | 0.046 |
| 1-8-2 Type of Employment (Base: Non-regular Employee) |  |  |  |  |  |  |  |  |
| Regular Employee | 0.018 | -0.004 | 0.004 | 0.008 | **-0.083**** | -0.069* | **-0.080**** | **-0.084**** |
| 1-8-3 Type of Practice |  |  |  |  |  |  |  |  |
| Specialized Clinical Pharmacist (Base: General) | 0.023 | -0.014 | -0.030 | 0.013 | -0.027 | -0.070* | -0.060 | -0.045 |
| Full-time Clinical Pharmacist (Base: Part-time) | 0.060 | 0.056 | 0.065 | 0.056 | -0.068* | 0.008 | -0.015 | -0.020 |
| 1-9-1 Terminal Degree (Base: Junior College Degree or below) |  |  |  |  |  |  |  |  |
| Bachelor Degree | -0.130 | -0.116 | -0.026 | -0.065 | 0.204 | 0.220 | 0.144 | 0.206 |
| Master Degree | -0.105 | -0.049 | 0.009 | -0.024 | 0.259 | 0.250 | 0.185 | 0.254* |
| Doctor Degree or above | -0.005 | 0.003 | 0.022 | 0.025 | 0.216* | 0.209* | 0.169 | **0.212**** |
| 1-9-2 Major (Base: Clinical Pharmacy) |  |  |  |  |  |  |  |  |
| Other Pharmacy-allied Majors | 0.028 | 0.074 | 0.044 | 0.040 |  |  |  |  |
| Non-pharmaceutical-related Majors | 0.045 | 0.013 | 0.015 | 0.039 |  |  |  |  |
| 1-9-3 Pattern of Training (Base: Training after Graduation) |  |  |  |  |  |  |  |  |
| Training after Job Transfer | 0.029 | -0.001 | -0.052 | 0.024 |  |  |  |  |
| Direct Assignment without Training | **-0.103**** | **-0.116**** | **-0.140***** | -0.078* |  |  |  |  |
| 1-10-1 Interdisciplinary Education Experience |  |  |  |  |  |  |  |  |
| Have Taken Basic Medical/Pharmaceutical Courses (Base: No) | -0.071* | -0.057 | -0.069 | **-0.102**** | -0.028 | **-0.086**** | -0.033 | -0.057 |
| Have Taken Management Science Courses (Base: No) | 0.077 | 0.001 | 0.037 | 0.046 | 0.002 | 0.009 | -0.021 | 0.007 |
| 1-10-2 Inter-career Working Experience (Base: No) | 0.082 | 0.034 | 0.061 | 0.061 | -0.006 | 0.031 | -0.027 | 0.004 |
| 1-10-3 Interprofessional Collaboration Experience | -0.051 | 0.025 | 0.076 | 0.012 | -0.039 | -0.011 | -0.005 | -0.012 |
| 1-11 Personality |  |  |  |  |  |  |  |  |
| 1-11-1 Extraversion | 0.014 | -0.011 | -0.003 | 0.005 | 0.051 | 0.054 | 0.029 | 0.064 |
| 1-11-2 Agreeableness | 0.068 | 0.004 | 0.060 | 0.029 | 0.018 | 0.024 | -0.023 | 0.016 |
| 1-11-3 Conscientiousness | **0.108***** | **0.119**** | **0.174***** | **0.111**** | -0.080* | **-0.111**** | -0.041 | **-0.087**** |
| 1-11-4 Neuroticism | -0.014 | -0.006 | -0.056 | 0.010 | 0.014 | 0.040 | 0.003 | 0.009 |
| 1-11-5 Openness | 0.049 | 0.067 | 0.038 | 0.073* | 0.015 | 0.030 | 0.007 | 0.028 |
| 2-1 Institution Administration |  |  |  |  |  |  |  |  |
| 2-1-1 System Construction | 0.122* | -0.002 | 0.000 | 0.102* | **0.255***** | **0.203***** | **0.179***** | **0.240***** |
| 2-1-2 Manager Support | 0.085 | **0.256***** | 0.087 | **0.210***** | 0.021 | **0.245***** | **0.184***** | **0.165***** |
| 2-1-3 Personnel Development | -0.013 | -0.037 | 0.043 | -0.024 | 0.027 | 0.010 | 0.031 | 0.030 |
| 2-2 Job Satisfaction |  |  |  |  |  |  |  |  |
| 2-2-1 Workload | 0.048 | 0.018 | 0.067 | 0.022 | 0.018 | -0.011 | -0.039 | -0.022 |
| 2-2-2 Working Environment | 0.064 | 0.031 | 0.046 | 0.039 | 0.051 | 0.031 | 0.006 | 0.034 |
| 2-2-3 Remuneration | 0.045 | 0.056 | 0.115* | 0.011 | **0.123**** | 0.041 | 0.069 | 0.032 |
| 2-3 Working Atmosphere |  |  |  |  |  |  |  |  |
| 2-3-1 Team Acceptability | 0.103** | 0.001 | 0.076* | -0.005 | 0.011 | -0.052 | -0.015 | -0.018 |
| 2-3-2 Social Acceptability | 0.011 | 0.074 | 0.019 | 0.052 | -0.055 | -0.019 | -0.041 | -0.024 |
| 2-4 Resources and Conditions |  |  |  |  |  |  |  |  |
| 2-4-1 Adequate Personnel Allocation (Base: No) | -0.063 | -0.032 | -0.037 | -0.058 | -0.020 | 0.002 | **-0.084**** | -0.029 |
| 2-4-2 Numbers of Needed Facilities and Equipment | 0.012 | -0.008 | 0.057 | -0.019 | -0.030 | -0.029 | -0.045 | -0.051 |
| 2-5 Collaboration Incentives (Base: No) | **-0.132**** | -0.038 | -0.083* | -0.069 | 0.005 | -0.015 | 0.011 | 0.001 |
| 3-1 Respect | 0.062 | **0.207***** | 0.053 | 0.124* | -0.117 | -0.096 | -0.091 | -0.113* |
| 3-2 Trust | 0.074 | -0.023 | 0.004 | 0.051 | **0.176***** | **0.164***** | 0.116 | **0.165***** |
| 3-3 Role Recognition | -0.061 | -0.018 | **0.134**** | 0.027 | 0.044 | 0.005 | 0.014* | 0.031 |
| 3-4 Fairness | -0.084 | -0.049 | -0.100 | -0.040 | **-0.199***** | **-0.159***** | -0.105* | **-0.13**** |
| 3-5 Attitude to Collaboration |  |  |  |  |  |  |  |  |
| 3-5-1 Job Burnout | 0.020 | 0.057 | 0.040 | 0.019 | 0.003 | 0.016 | 0.024 | 0.017 |
| 3-5-2 Usefulness Perception | 0.049 | 0.027 | 0.037 | 0.031 | **0.127***** | **0.128***** | **0.185***** | **0.141***** |
| 3-5-3 Shared Expectation | 0.107* | **0.108**** | 0.089 | **0.144***** | **0.149***** | **0.095**** | **0.130***** | **0.126***** |
| 3-6 Leadership |  |  |  |  |  |  |  |  |
| 3-6-1 Power Influence (Base: Same Office Rank) |  |  |  |  |  |  |  |  |
| Higher Rank of Physician | -0.063 | -0.069 | 0.011 | -0.057 | -0.078* | **-0.125***** | -0.060 | **-0.103**** |
| Higher Rank of Clinical Pharmacist | -0.005 | 0.049 | -0.016 | 0.015 | -0.017 | 0.004 | -0.001 | -0.004 |
| 3-6-2 Non-Power Influence |  |  |  |  |  |  |  |  |
| Effect of Morality and Sentiment (Base: No) | 0.087* | 0.072* | 0.073* | 0.073* | 0.021 | 0.034 | 0.040 | 0.032 |
| Effect of Knowledge and Ability (Base: No) | 0.051 | 0.023 | 0.026 | 0.045 | -0.015 | -0.006 | -0.007 | -0.002 |
| 3-7 Familiarity |  |  |  |  |  |  |  |  |
| 3-7-1 Member Mobility | -0.013 | -0.024 | -0.066 | -0.028 | -0.005 | 0.025 | -0.022 | -0.004 |
| 3-7-2 Out-of-Work Socializing | -0.038 | 0.019 | -0.064 | -0.003 | -0.001 | 0.023 | 0.003 | 0.016 |
| 3-8 Communication |  |  |  |  |  |  |  |  |
| 3-8-1 Team Communication Ability | 0.033 | 0.020 | 0.108 | 0.012 | 0.067 | -0.058 | **0.157**** | 0.072 |
| 3-8-2 General Language Ability | **0.174**** | 0.113* | 0.120* | **0.150**** | **0.177**** | **0.213***** | 0.058 | **0.159**** |

Note:

1.*,**,*** indicate respectively P＜0.1，P＜0.05，P＜0.0.1. Variables with P>0.05 are bolded to facilitate reading;

2. The blank part in the columns of “physician” indicates that this factor is only measured for clinical pharmacists. See Supplement 4 for the complete questionnaire.

3. two approach of robustness analysis are adopted in this table: (1) subdivision of explained variables: to test the consistency of regression results of each independent variable on the three dimensions of PPCI, namely trustworthiness, role perception and relationship initiation (columns (1), (2) and (3)); (2) change of sample size: to eliminate adverse effect of outliers on regression results by winsorizing on 1% and 99% quantiles for continuous variables in the sample.(Column (4)).

**Supplement 4: Questionnaire - Clinical Pharmacist Version**

(The differences in the physician version of the questionnaire are explained in brackets in the corresponding items.)

| **No.** | **Questions** | **Options** | | |
| --- | --- | --- | --- | --- |
| **1** | **Basic Information** | | | |
| **1.1** | Gender | □Male □Female | | |
| **1.2** | Age | Years old | | |
| **1.3** | Marital status | □Married □Unmarried □Other (divorced, widowed, etc.) | | |
| **1.4** | Years of clinical practice | Years | | |
| **1.5** | Location | _________ (province/autonomous region/ municipality) | | |
| **1.6** | Which of the following clinical departments are you mainly involved in? (Multiple options) | □General Medicine □Respiratory Medicine □Hematology □Nephrology □Endocrinology □Neurology  □Gastroenterology □Cardiovascular Medicine □Oncology □Surgery □Obstetrics and Gynecology  □Emergency Medicine □Psychiatry □Immunization □Pediatrics □Infection □Pain □Critical Care  □Anesthesiology □Traditional Chinese Medicine □Rehabilitation □Dermatology □Stomatology  □ Ophthalmology □Otorhinolaryngology □Geriatrics □Others________ | | |
| **1.7** | The grade of the hospital you are working in | □Tertiary Hospital □Secondary Hospital □Others | | |
| **1.8** | The type of the hospital you are working in | □General Hospital □Specialized Hospital □Others | | |
| **1.9** | Is your hospital an authorized training base for clinical pharmacists? | □Yes □No | | |
| **1.10** | Professional title | □Junior Title □Intermediate Title □Associate Senior Title □Senior Title | | |
| **1.11** | Type of employment | □Regular Employee □Non-regular Employee | | |
| **1.12** | Type of clinical pharmacist  (This item is excluded in physician version) | □Full-time Clinical Pharmacist □Part-time Clinical Pharmacist  □Specialist Clinical Pharmacist □General Clinical Pharmacist | | |
| **1.13** | Terminal degree | □Junior College Degree or below □Bachelor Degree □Master Degree □Doctor Degree or above | | |
| **1.14** | Major  (This item is excluded in physician version) | □Clinical Pharmacy  □Pharmacy (Pharmacology/Pharmacy/Pharmaceutical Chemistry/Pharmaceutical Analysis)  □Other pharmacy-related majors  □Medical and nursing majors  □Non-related majors | | |
| **1.16** | Pattern of training  (This item is excluded in physician version) | □Training after Graduation  □Training after Job Transfer  □Direct Assignment without Training  □Others________ | | |
|  | | | | |
| **2** | **PPCI** | | | |
| **2.1** | This physician is credible. | □Disagree strongly □Disagree □Disagree a little □Unsure □Agree a little □Agree □Agree strongly | | |
| **2.2** | I trust this physician. | □Disagree strongly □Disagree □Disagree a little □Unsure □Agree a little □Agree □Agree strongly | | |
| **2.3** | I can count on this physician to do what he/she says. | □Disagree strongly □Disagree □Disagree a little □Unsure □Agree a little □Agree □Agree strongly | | |
| **2.4** | Communication between this physician and myself is two-way. | □Disagree strongly □Disagree □Disagree a little □Unsure □Agree a little □Agree □Agree strongly | | |
| **2.5** | I intend to keep working together with this physician. | □Disagree strongly □Disagree □Disagree a little □Unsure □Agree a little □Agree □Agree strongly | | |
| **2.6** | My interactions with this physician are characterized by open communication of both parties. | □Disagree strongly □Disagree □Disagree a little □Unsure □Agree a little □Agree □Agree strongly | | |
| **2.7** | This physician and I negotiate to come to an agreement on my activities in managing drug therapy. | □Disagree strongly □Disagree □Disagree a little □Unsure □Agree a little □Agree □Agree strongly | | |
| **2.8** | This physician and I are mutually dependent on each other in caring for patients. | □Disagree strongly □Disagree □Disagree a little □Unsure □Agree a little □Agree □Agree strongly | | |
| **2.9** | This physician will work with me to overcome disagreements on my role in managing drug therapy. | □Disagree strongly □Disagree □Disagree a little □Unsure □Agree a little □Agree □Agree strongly | | |
| **2.10** | For our practices, I need this physician as much as this physician needs me. | □Disagree strongly □Disagree □Disagree a little □Unsure □Agree a little □Agree □Agree strongly | | |
| **2.11** | This physician depends on me as much as I depend on him/her. | □Disagree strongly □Disagree □Disagree a little □Unsure □Agree a little □Agree □Agree strongly | | |
| **2.12** | I spend time trying to learn how I can help this physician provide better care. | □Disagree strongly □Disagree □Disagree a little □Unsure □Agree a little □Agree □Agree strongly | | |
| **2.13** | I show an interest in helping this physician improve his/her practice. | □Disagree strongly □Disagree □Disagree a little □Unsure □Agree a little □Agree □Agree strongly | | |
| **2.14** | I provide information to this physician about specific patients. | □Disagree strongly □Disagree □Disagree a little □Unsure □Agree a little □Agree □Agree strongly | | |
|  | | | | |
| **3.1** | Have you taken the following courses? | | | |
|  | Basic Medical (Pharmaceutical) Courses | □Yes □No | | |
|  | Management Science Courses | □Yes □No | | |
| **3.2** | Do you have cross-career work experience (e.g. as a physician, nurse, etc.)? | □Yes □No | | |
| **3.3** | Have you worked with other medical personnel (e.g. nurses, dietitians, therapists, psychological counselors, social workers, etc.) before working with physicians? |  | | |
|  | (1) Joint ward round and case discussion | □Yes □No | | |
|  | (2) Joint participation in multidisciplinary consultations | □Yes □No | | |
|  | (3) Participate in patient diagnosis and treatment and adverse event treatment together | □Yes □No | | |
|  | (4) Jointly carry out patient consultation and education | □Yes □No | | |
|  | (5) Negotiate and modify unreasonable prescriptions | □Yes □No | | |
|  | (6) To participate in professional training or conferences together | □Yes □No | | |
|  | (7) Jointly carry out research projects | □Yes □No | | |
|  | (8) Others**_______________________________________** | | | |
|  | | | | |
| **4** | **How well do the following statements describe your personality?** | | | |
| **4.1** | … is reserved | □Disagree strongly □Disagree a little □Unsure □Agree a little □Agree strongly | | |
| **4.2** | … is generally trusting | □Disagree strongly □Disagree a little □Unsure □Agree a little □Agree strongly | | |
| **4.3** | … tends to be lazy | □Disagree strongly □Disagree a little □Unsure □Agree a little □Agree strongly | | |
| **4.4** | … is relaxed, handles stress well | □Disagree strongly □Disagree a little □Unsure □Agree a little □Agree strongly | | |
| **4.5** | … has few artistic interests | □Disagree strongly □Disagree a little □Unsure □Agree a little □Agree strongly | | |
| **4.6** | … is outgoing, sociable | □Disagree strongly □Disagree a little □Unsure □Agree a little □Agree strongly | | |
| **4.7** | … tends to find fault with others | □Disagree strongly □Disagree a little □Unsure □Agree a little □Agree strongly | | |
| **4.8** | … does a thorough job | □Disagree strongly □Disagree a little □Unsure □Agree a little □Agree strongly | | |
| **4.9** | … gets nervous easily | □Disagree strongly □Disagree a little □Unsure □Agree a little □Agree strongly | | |
| **4.10** | … has an active imagination | □Disagree strongly □Disagree a little □Unsure □Agree a little □Agree strongly | | |
|  | | | | |
| **5.** | **How well do the following statements describe the administration of the hospital you are working in?** | | | |
| **5.1** | The hospital has clear regulations on the work flow of clinical pharmacists | | | □Disagree strongly □Disagree a little □Unsure □Agree a little □Agree strongly |
| **5.2** | The hospital has clear regulations on the cooperative working mode and responsibilities of clinical pharmacists and physicians | | | □Disagree strongly □Disagree a little □Unsure □Agree a little □Agree strongly |
| **5.3** | The organization of clinical pharmacists in the hospital is reasonable | | | □Disagree strongly □Disagree a little □Unsure □Agree a little □Agree strongly |
| **5.4** | The hospital's performance appraisal system for clinical pharmacists is fair and reasonable | | | □Disagree strongly □Disagree a little □Unsure □Agree a little □Agree strongly |
| **5.5** | The promotion mechanism of the hospital is fair and reasonable | | | □Disagree strongly □Disagree a little □Unsure □Agree a little □Agree strongly |
| **5.6** | Managers attach great importance to the construction of collaborative working mode between clinical pharmacists and physicians | | | □Disagree strongly □Disagree a little □Unsure □Agree a little □Agree strongly |
| **5.7** | Clinical pharmacists can be supported for their innovative ideas in their work | | | □Disagree strongly □Disagree a little □Unsure □Agree a little □Agree strongly |
| **5.8** | Clinical pharmacists have the opportunity to participate in the development of management policy | | | □Disagree strongly □Disagree a little □Unsure □Agree a little □Agree strongly |
| **5.9** | Management can publicly acknowledge the contributions made by clinical pharmacists | | | □Disagree strongly □Disagree a little □Unsure □Agree a little □Agree strongly |
| **5.10** | Managers have no obvious emotional tendency in management | | | □Disagree strongly □Disagree a little □Unsure □Agree a little □Agree strongly |
| **5.11** | The hospital has a complete training system for clinical pharmacists | | | □Disagree strongly □Disagree a little □Unsure □Agree a little □Agree strongly |
| **5.12** | My personal abilities and strengths have been given full play | | | □Disagree strongly □Disagree a little □Unsure □Agree a little □Agree strongly |
| **5.13** | On-the-job training and continuing education are supported | | | □Disagree strongly □Disagree a little □Unsure □Agree a little □Agree strongly |
| **5.14** | The hospital can provide me with the professional training and information I need | | | □Disagree strongly □Disagree a little □Unsure □Agree a little □Agree strongly |
| **5.15** | Hospitals can proactively provide cross-professional skills training | | | □Disagree strongly □Disagree a little □Unsure □Agree a little □Agree strongly |
|  | | | | |
| **6** | **Resources and Conditions** | | | |
| **6.1** | Whether the current clinical pharmacist staffing can meet the clinical needs? | | □Yes □No | |
| **6.2** | Is the hospital able to provide you with the use of the following software systems? (Multiple options) | | □Hospital Information system □Electronic medical record system  □Monitoring system for rational drug use □Prescription review system  □Clinical pharmacist workstation □ Clinical medication support system  □ Others ___________________ | |
| **6.3** | Is the hospital able to provide you with the use of the following facilities? (Multiple options)  (This item is not included in physician version) | | □Library room □ Pharmaceutical Information Room/Internet  □Intravenous Drug Dispensing Center □Prescription Examination Center  □Pharmaceutical Clinic □ Drug Store □Quality Control Room □Others_______________ | |
|  | | | | |
| **7** | **Job satisfaction** | | | |
| **7.1** | You are stressed about your career. | | □Disagree strongly □Disagree a little □Unsure □Agree a little □Agree strongly | |
| **7.2** | You have too much work for you alone | | □Disagree strongly □Disagree a little □Unsure □Agree a little □Agree strongly | |
| **7.3** | You feel comfortable in your work environment | | □Disagree strongly □Disagree a little □Unsure □Agree a little □Agree strongly | |
| **7.4** | You feel convenient in your work | | □Disagree strongly □Disagree a little □Unsure □Agree a little □Agree strongly | |
| **7.5** | You are satisfied with your salary | | □Disagree strongly □Disagree a little □Unsure □Agree a little □Agree strongly | |
| **7.6** | You are satisfied with the payment of overtime wages and benefits | | □Disagree strongly □Disagree a little □Unsure □Agree a little □Agree strongly | |
| **7.7** | Does your institution have additional incentives for clinical pharmacists to collaborate with physicians? | | □Yes □No | |
|  | | | | |
| **8** | **How well do the following statements describe the atmosphere of the collaboration team you are involved in?** | | | |
| **8.1** | Team members communicate openly and directly | | □Disagree strongly □Disagree a little □Unsure □Agree a little □Agree strongly | |
| **8.2** | Team members ask tough questions | | □Disagree strongly □Disagree a little □Unsure □Agree a little □Agree strongly | |
| **8.3** | Team members don't offer unsolicited advice | | □Disagree strongly □Disagree a little □Unsure □Agree a little □Agree strongly | |
| **8.4** | There was open opposition to the goals set for the team | | □Disagree strongly □Disagree a little □Unsure □Agree a little □Agree strongly | |
| **8.5** | It is difficult for team members to ask others for help | | □Disagree strongly □Disagree a little □Unsure □Agree a little □Agree strongly | |
| **8.6** | It's okay to make mistakes on this team | | □Disagree strongly □Disagree a little □Unsure □Agree a little □Agree strongly | |
| **8.7** | You need to be careful about how you get along with your team members | | □Disagree strongly □Disagree a little □Unsure □Agree a little □Agree strongly | |
| **8.8** | When a team member makes a mistake, the rest of the team takes issue with it | | □Disagree strongly □Disagree a little □Unsure □Agree a little □Agree strongly | |
|  | | | | |
| **How well do the following statements describe the social atmosphere of the occupation of clinical pharmacist (physician)?** | | | | |
| **8.9** | The understanding and support of my colleagues can make me feel happy | | □Disagree strongly □Disagree a little □Unsure □Agree a little □Agree strongly | |
| **8.10** | A career as a clinical pharmacist would make it easier for me to gain the respect of the public | | □Disagree strongly □Disagree a little □Unsure □Agree a little □Agree strongly | |
| **8.11** | I get good feedback from patients and families | | □Disagree strongly □Disagree a little □Unsure □Agree a little □Agree strongly | |
| **8.12** | I feel a sense of social responsibility to continue my career as a clinical pharmacist | | □Disagree strongly □Disagree a little □Unsure □Agree a little □Agree strongly | |
| **8.13** | Patients and their families have a good recognition of clinical pharmacists | | □Disagree strongly □Disagree a little □Unsure □Agree a little □Agree strongly | |
|  |  | | | |
| **9** | **Trust and respect** | | | |
| **9.1** | Clinical pharmacists have been recognized for their professional competence in the hospital | | □Disagree strongly □Disagree a little □Unsure □Agree a little □Agree strongly | |
| **9.2** | Clinical pharmacists are respected by physicians | | □Disagree strongly □Disagree a little □Unsure □Agree a little □Agree strongly | |
| **9.3** | The independence of clinical pharmacists is guaranteed | | □Disagree strongly □Disagree a little □Unsure □Agree a little □Agree strongly | |
| **9.4** | The decision-making body in the hospital includes the clinical pharmacist organization | | □Disagree strongly □Disagree a little □Unsure □Agree a little □Agree strongly | |
| **For the physicians (clinical pharmacists) you work with:** | | | | |
| **9.5** | He/she is a person who takes the collaborative work of clinical pharmacists and physicians seriously | | □Disagree strongly □Disagree a little □Unsure □Agree a little □Agree strongly | |
| **9.6** | He/she is willing to make important contributions to the clinical pharmacist/physician collaboration team | | □Disagree strongly □Disagree a little □Unsure □Agree a little □Agree strongly | |
| **9.7** | You can rely on him/her to do a major part of the team's work | | □Disagree strongly □Disagree a little □Unsure □Agree a little □Agree strongly | |
| **9.8** | He/she is the one who can work with a collaborative team | | □Disagree strongly □Disagree a little □Unsure □Agree a little □Agree strongly | |
|  |  | |  | |
| **10** | **Role Recognition** | | | |
| **10.1** | I know what my and physicians’ responsibilities and rights are | | | □Disagree strongly □Disagree a little □Unsure □Agree a little □Agree strongly |
| **10.2** | I am well aware of what is expected of me in my job/organization | | | □Disagree strongly □Disagree a little □Unsure □Agree a little □Agree strongly |
| **10.3** | I've managed my time reasonably | | | □Disagree strongly □Disagree a little □Unsure □Agree a little □Agree strongly |
| **10.4** | I have clear plans and goals of my work | | | □Disagree strongly □Disagree a little □Unsure □Agree a little □Agree strongly |
|  | | | | |
| **11** | **How well do the following statements describe your attitude to your work and collaboration?** | | | |
| **11.1** | Since I started this job, I have become less and less interested in my work | | | □Never □Several times a year □Once a month □Several times a month □Once a week □Several times a week □Every day |
| **11.2** | I'm not as enthusiastic about my work as I used to be | | | □Never □Several times a year □Once a month □Several times a month □Once a week □Several times a week □Every day |
| **11.3** | I doubted the meaning of what I was doing | | | □Never □Several times a year □Once a month □Several times a month □Once a week □Several times a week □Every day |
| **11.4** | I became less and less concerned about whether I was contributing to the work I was doing | | | □Never □Several times a year □Once a month □Several times a month □Once a week □Several times a week □Every day |
| **11.5** | I think it's useful for clinical pharmacists to work with physicians | | | □Never □Several times a year □Once a month □Several times a month □Once a week □Several times a week □Every day |
| **11.6** | I am confident in working with physicians to improve the quality of drug treatment for patients | | | □Disagree strongly □Disagree □Disagree a little □Unsure □Agree a little □Agree □Agree strongly |
| **11.7** | Physicians and I always adhere to the patient-centered, to improve the level of rational drug use as the expectation of cooperation | | | □Disagree strongly □Disagree □Disagree a little □Unsure □Agree a little □Agree □Agree strongly |
|  | | | | |
| **12** | **Leadership** | | | |
| **12.1** | Are you on the same administrative rank as a physician? | | | □Higher Rank of Physician □Same Office Rank □Higher Rank of Clinical Pharmacist |
| **12.2** | What do you think is the most attractive non-power influence for a physician to work with? (Multiple options) | | | □Moral character (morality, conduct, work style, value orientation, etc.)  □Knowledge (advanced thinking, talent, knowledge, etc.)  □Ability (organization, management, innovation, interpersonal skills, etc.)  □Affection (kindness, care, love, respect, etc.) |
|  | | | | |
| **13** | **How well do the following statements describe your (clinical pharmacist’s) communication ability?** | | | |
| **13.1** | Take the initiative to communicate with the doctor in time to ensure the accuracy and rationality of the doctor's orders | | | □Very bad □Bad □Unsure □Good □Very Good |
| **13.2** | Report patient's condition changes and drug needs to the doctor without delay | | | □Very bad □Bad □Unsure □Good □Very Good |
| **13.3** | Be able to communicate effectively with the doctor in charge on the patient's medication and participate in decision making | | | □Very bad □Bad □Unsure □Good □Very Good |
| **13.4** | Give timely and accurate answers to the doctor's drug information consultation | | | □Very bad □Bad □Unsure □Good □Very Good |
| **13.5** | Greet the others when I see them | | | □Very bad □Bad □Unsure □Good □Very Good |
| **13.6** | Use friendly nouns to address each other | | | □Very bad □Bad □Unsure □Good □Very Good |
| **13.7** | Pay attention to the speed of speech, so that the other party can hear the content clearly | | | □Very bad □Bad □Unsure □Good □Very Good |
| **13.8** | Pay attention to the tone of voice when talking to make the other person feel comfortable | | | □Very bad □Bad □Unsure □Good □Very Good |
| **13.9** | Pay attention to the other person's language habits and choose the right words | | | □Very bad □Bad □Unsure □Good □Very Good |
| **13.10** | Use polite language in conversation | | | □Very bad □Bad □Unsure □Good □Very Good |
| **13.11** | Communicate patiently with each other even if the work is busy | | | □Very bad □Bad □Unsure □Good □Very Good |
| **13.12** | When the other party asks me about the problem that I cannot solve, I can take the initiative to introduce other suitable consultation objects or channels | | | □Very bad □Bad □Unsure □Good □Very Good |
| **13.13** | Use humor appropriately when talking to create a good atmosphere | | | □Very bad □Bad □Unsure □Good □Very Good |
|  | | | | |
| **14** | **Fairness** | | | |
| **14.1** | My pay is in line with my performance | | □Disagree strongly □Disagree a little □Unsure □Agree a little □Agree strongly | |
| **14.2** | I think my current salary is commensurate with my workload | | □Disagree strongly □Disagree a little □Unsure □Agree a little □Agree strongly | |
| **14.3** | In collaborative work, my contribution is proportional to the feedback from the physician (clinical pharmacist) | | □Disagree strongly □Disagree a little □Unsure □Agree a little □Agree strongly | |
|  | | | | |
| **15** | **Familiarity** | | | |
| **15.1** | How often you collaborate with the physician (clinical pharmacist) to provide services to the same patient? | | □ Rarely □ Occasionally □ Common □ More □ Very much | |
| **15.2** | During holidays or after office hours, I would call or visit him/her. | | □Disagree strongly □Disagree a little □Unsure □Agree a little □Agree strongly | |
| **15.3** | He/her invites me to his/her home for lunch or dinner. | | □Disagree strongly □Disagree a little □Unsure □Agree a little □Agree strongly | |
| **15.4** | On special occasions such as birthday, I would definitely send him/her gifts. | | □Disagree strongly □Disagree a little □Unsure □Agree a little □Agree strongly | |
| **15.5** | I always actively share with him/her about my thoughts, problems, needs and  feelings. | | □Disagree strongly □Disagree a little □Unsure □Agree a little □Agree strongly | |
| **15.6** | I know his/her personal habits and hobbies. | | □Disagree strongly □Disagree a little □Unsure □Agree a little □Agree strongly | |
